# Author Correction: Statistical machine learning of sleep and physical activity phenotypes from sensor data in 96,220 UK Biobank participants

**DOI:** 10.1038/s41598-022-10148-5

**Published:** 2022-04-11

**Authors:** Matthew Willetts, Sven Hollowell, Louis Aslett, Chris Holmes, Aiden Doherty

**Affiliations:** 1 Department of Statistics, University of Oxford, Oxford, UK; 2 Big Data Institute, Li Ka Shing Centre for Health Information and Discovery, University of Oxford, Oxford, UK; 3 Nuffield Department of Population Health, BHF Centre of Research Excellence, University of Oxford, Oxford, UK; 4 Department of Mathematical Sciences, Durham University, Durham, UK; 5 Institute of Biomedical Engineering, Department of Engineering Science, University of Oxford, Oxford, UK

Correction to: *Scientific Reports* 10.1038/s41598-018-26174-1, published online: 21 May 2018

This Article contains an error in Supplementary Table 1, where the “Ground truth → Prediction↓” labels were incorrectly ordered as “Prediction → Ground truth↓”.

Additionally, the Data and code availability section is incomplete.

“Upon publication, the summary variables that we have constructed will be made available as a part of the UK Biobank dataset at http://biobank.ctsu.ox.ac.uk/crystal/label.cgi?id=1008. All data processing, feature extraction, machine learning, and analysis code will be available at https://github.com/activityMonitoring.”

should read:

“Upon publication, the summary variables that we have constructed are available as a part of the UK Biobank dataset at https://biobank.ndph.ox.ac.uk/ukb/dset.cgi?id=2242. All data processing, feature extraction, machine learning, and analysis code are available at https://github.com/activityMonitoring. The underlying data used to train the human activity recognition model in this paper is available at https://github.com/activityMonitoring/capture24.”

The corrected Supplementary Table 1 file is linked to this correction notice.

## Supplementary Information

Supplementary Information.

