# Supplementary material for: Statistical machine learning of sleep and physical activity phenotypes from sensor data in 96,220 UK Biobank participants
Source: Sci Rep. 2018 May 21;8:7961. doi: 10.1038/s41598-018-26174-1 (PMC5962537; doi:10.1038/s41598-018-26174-1)
Supplement: Supplementary file 1 — Supplementary tables and figures [file 41598_2018_26174_MOESM1_ESM.docx]

**Statistical machine learning of sleep and physical activity phenotypes from sensor data in 96,220 UK Biobank participants.**

**Matthew Willetts, Sven Hollowell, Louis Aslett, Chris Holmes, Aiden Doherty**

# Supplementary Tables / Figures

**Table. S1.** Differences between confusion matrices: machine learned behaviours classified from wrist-worn accelerometer data when using Hidden Markov Models versus not: the CAPTURE-24 study 2014-2015 (n = 132).

1. Random Forest Only *(unit = minutes of behaviour)*

| Prediction→  Ground truth↓ | **sleep** | **sit/stand** | **vehicle** | **walking** | **mixed-activity** | **bicycling** |
| --- | --- | --- | --- | --- | --- | --- |
| **sleep** | **65,906** | 3,087 | 22 | 17 | 28 | 13 |
| **sit/stand** | 6,389 | **37,917** | 833 | 1,162 | 1,616 | 160 |
| **vehicle** | 280 | 3,439 | **3,355** | 557 | 1,071 | 86 |
| **walking** | 151 | 1,697 | 128 | **6,959** | 2,343 | 61 |
| **mixed-activity** | 365 | 5,489 | 640 | 3,688 | **10,042** | 187 |
| **bicycling** | 75 | 176 | 78 | 193 | 359 | **944** |

*b) Random Forest + Hidden Markov Model (unit = minutes of behaviour)*

| Prediction→  Ground truth↓ | **sleep** | **sit/stand** | **vehicle** | **walking** | **mixed-activity** | **bicycling** |
| --- | --- | --- | --- | --- | --- | --- |
| **sleep** | **71,253** | 1,973 | 0 | 14 | 18 | 0 |
| **sit/stand** | 1,694 | **43,947** | 551 | 1,464 | 1,594 | 176 |
| **vehicle** | 13 | 697 | **3,967** | 180 | 484 | 12 |
| **walking** | 58 | 1,021 | 177 | **6,856** | 1,452 | 65 |
| **mixed-activity** | 135 | 4,151 | 351 | 3,883 | **11,699** | 158 |
| **bicycling** | 13 | 17 | 10 | 180 | 210 | **1,041** |

1. Difference *(unit = minutes of behaviour)*

| Prediction→  Ground truth↓ | **sleep** | **sit/stand** | **vehicle** | **walking** | **mixed-activity** | **bicycling** |
| --- | --- | --- | --- | --- | --- | --- |
| **sleep** | **5,347** | -1,114 | -22 | -3 | -10 | -13 |
| **sit/stand** | -4,695 | **6,030** | -282 | 302 | -22 | 16 |
| **vehicle** | -267 | -2,742 | **612** | -378 | -587 | -74 |
| **walking** | -93 | -677 | 50 | **-104** | -891 | 4 |
| **mixed-activity** | -231 | -1,339 | -289 | 195 | **1,657** | -29 |
| **bicycling** | -63 | -159 | -69 | -13 | -149 | **97** |

### **Fig. S1.** Mean-difference plots for each category matching overall accelerometer predicted time versus the wearable camera groundtruth: the CAPTURE-24 study 2014-2015 (n = 132)

| 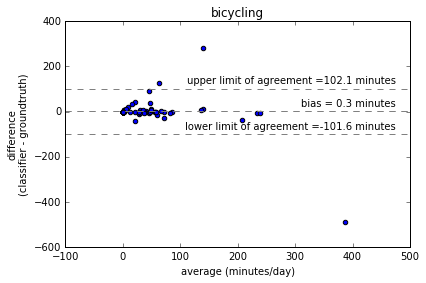 | 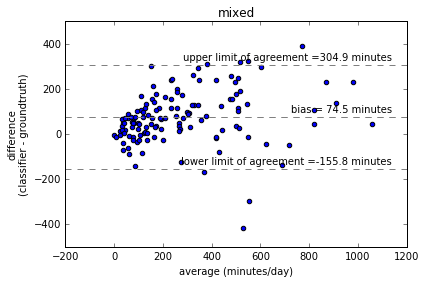 |
| --- | --- |
| 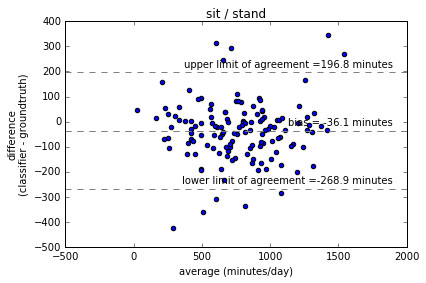 | 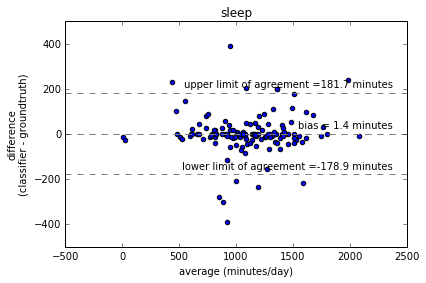 |
| 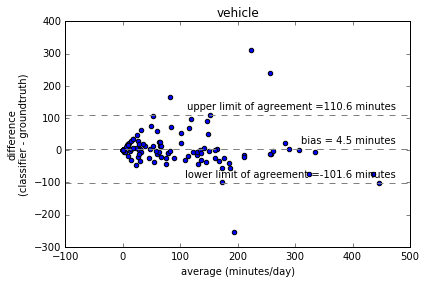 | 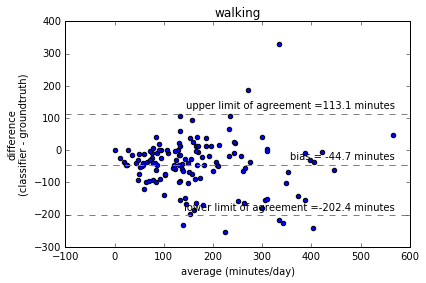 |
| 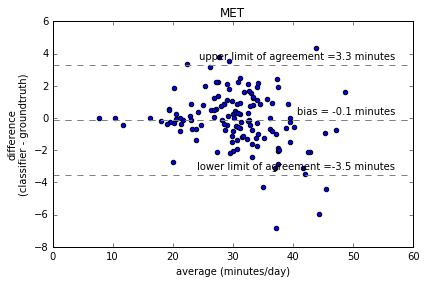 |  |

### **Fig. S2.** Feature importance measured by mean decrease in accuracy of the random forest classifier: the CAPTURE-24 study 2014-2015 (n = 132)


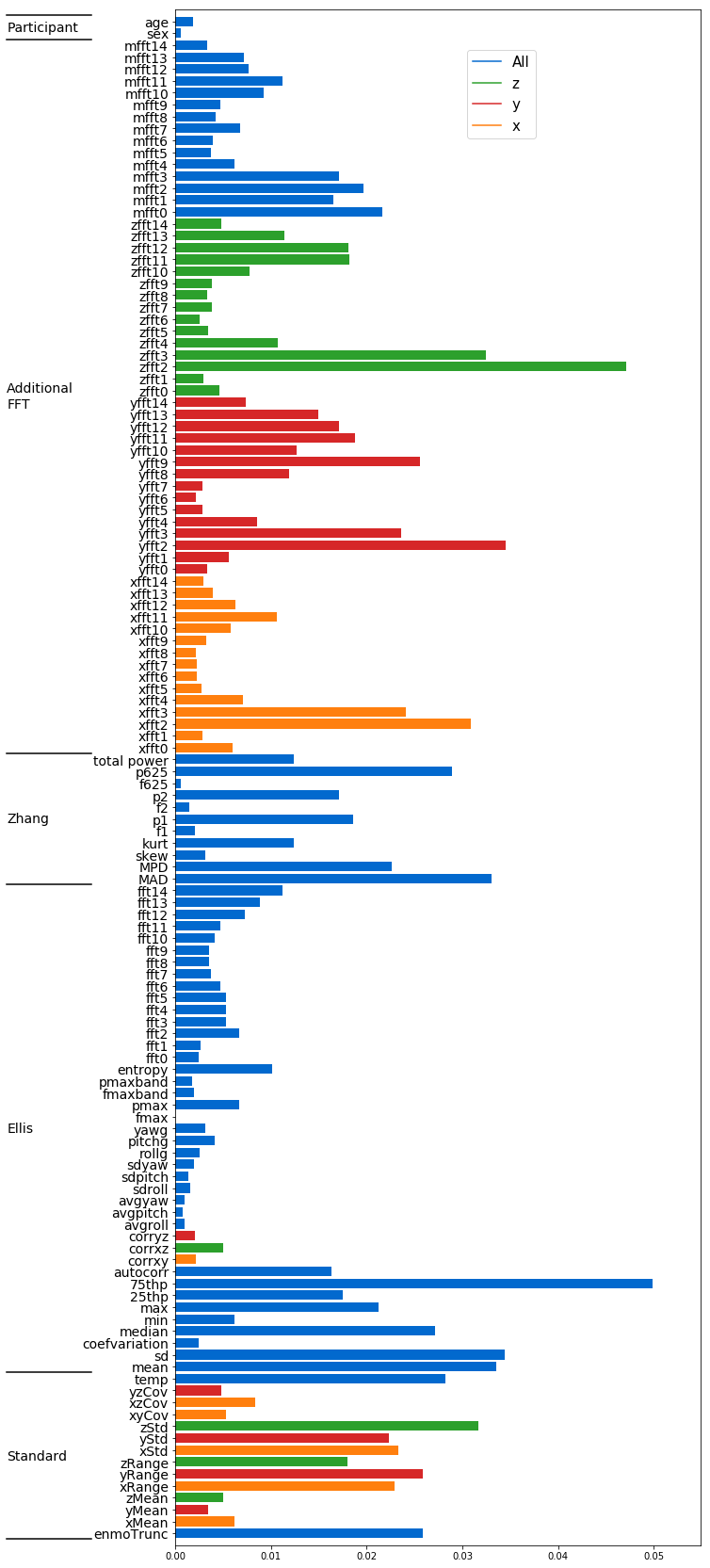


### **Fig. S3.** Variations in accuracy (kappa score) when increasing the number of decision trees in: unbalanced random forests (normal), balanced random forests, and when using Hidden Markov Models. The CAPTURE-24 study 2014-2015 (n = first 57 participants).


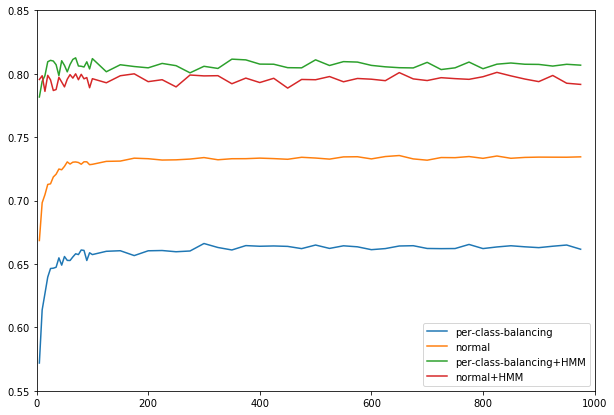


### **Fig. S4.** Variation in accelerometer-measured energy expenditure by activity type across weekdays and weekends: the UK Biobank study 2013-2015 (n = 96,220).


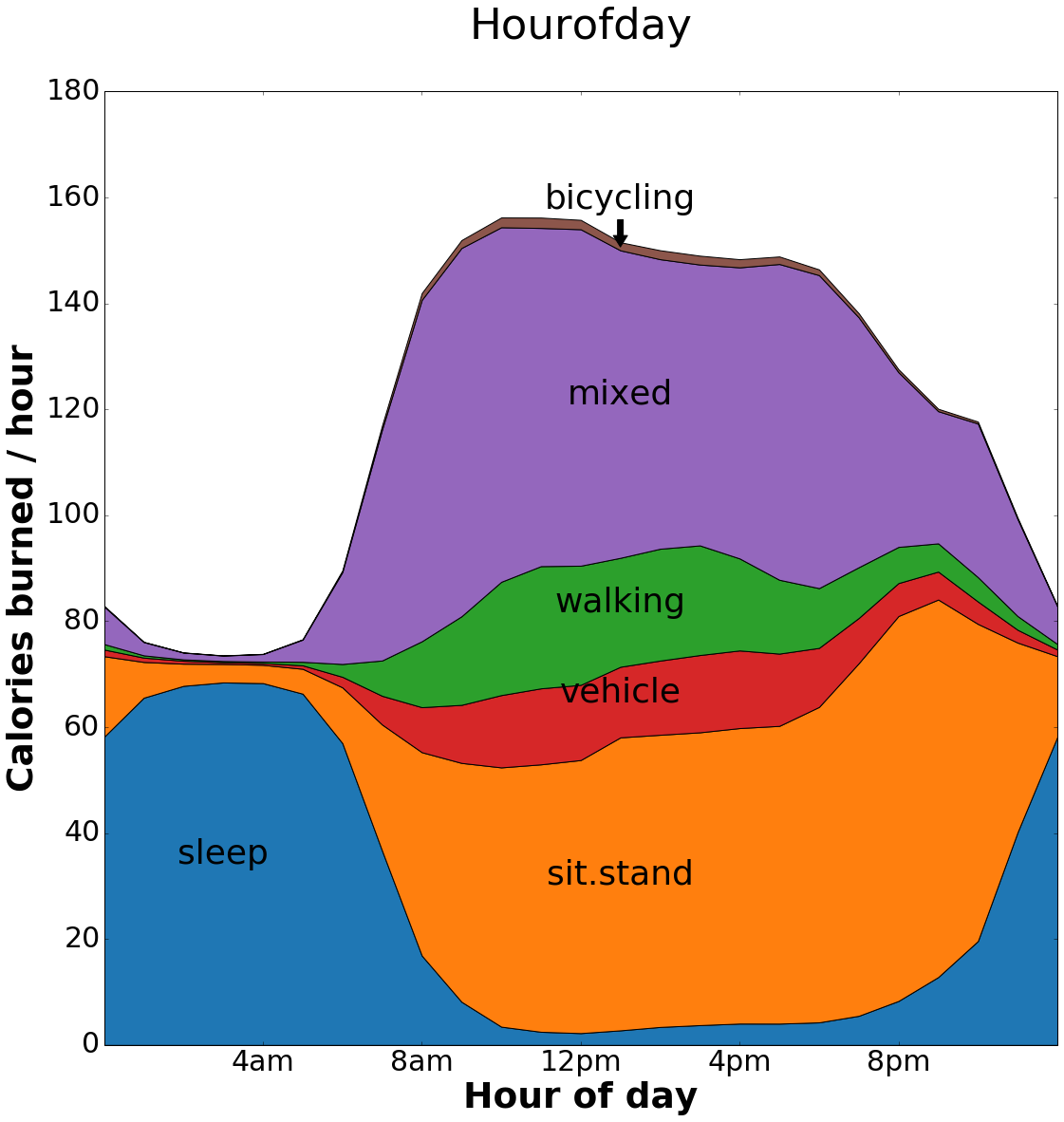


### **Fig. S5.** Correlation between accelerometer-measured vector magnitude, sleep, walking, sitting-or-standing, bicycling, vehicle, and mixed activity time: the UK Biobank study 2013-2015 (n = 96,220).


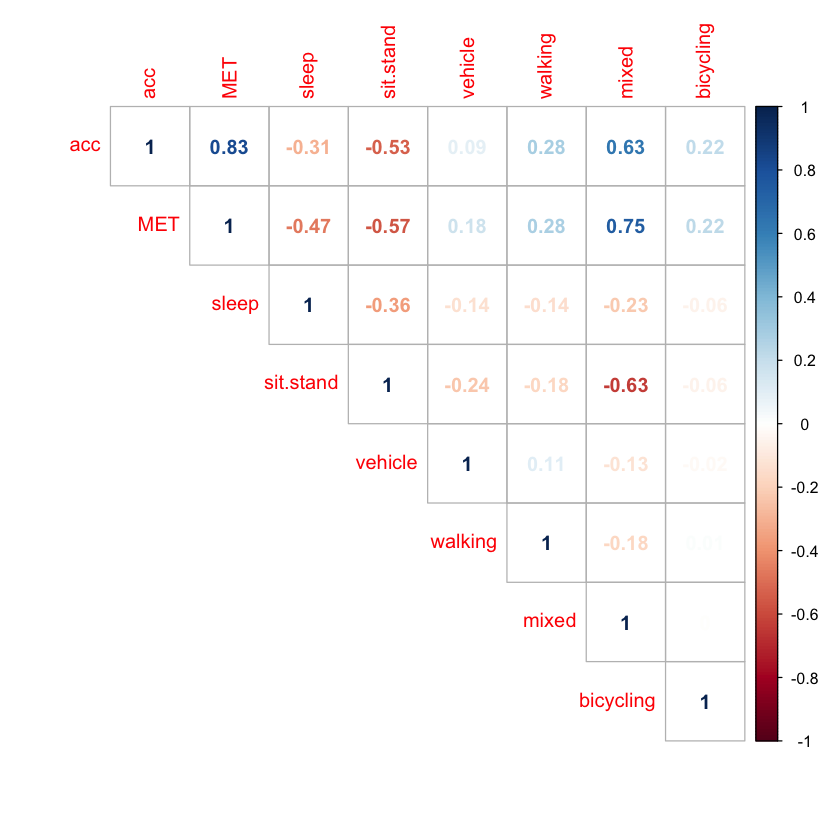


### **Fig. S6.** Visual representation of structure of free-living training set data, with labels colour coded: the CAPTURE-24 study 2014-2015 (n = first 57 participants).

###
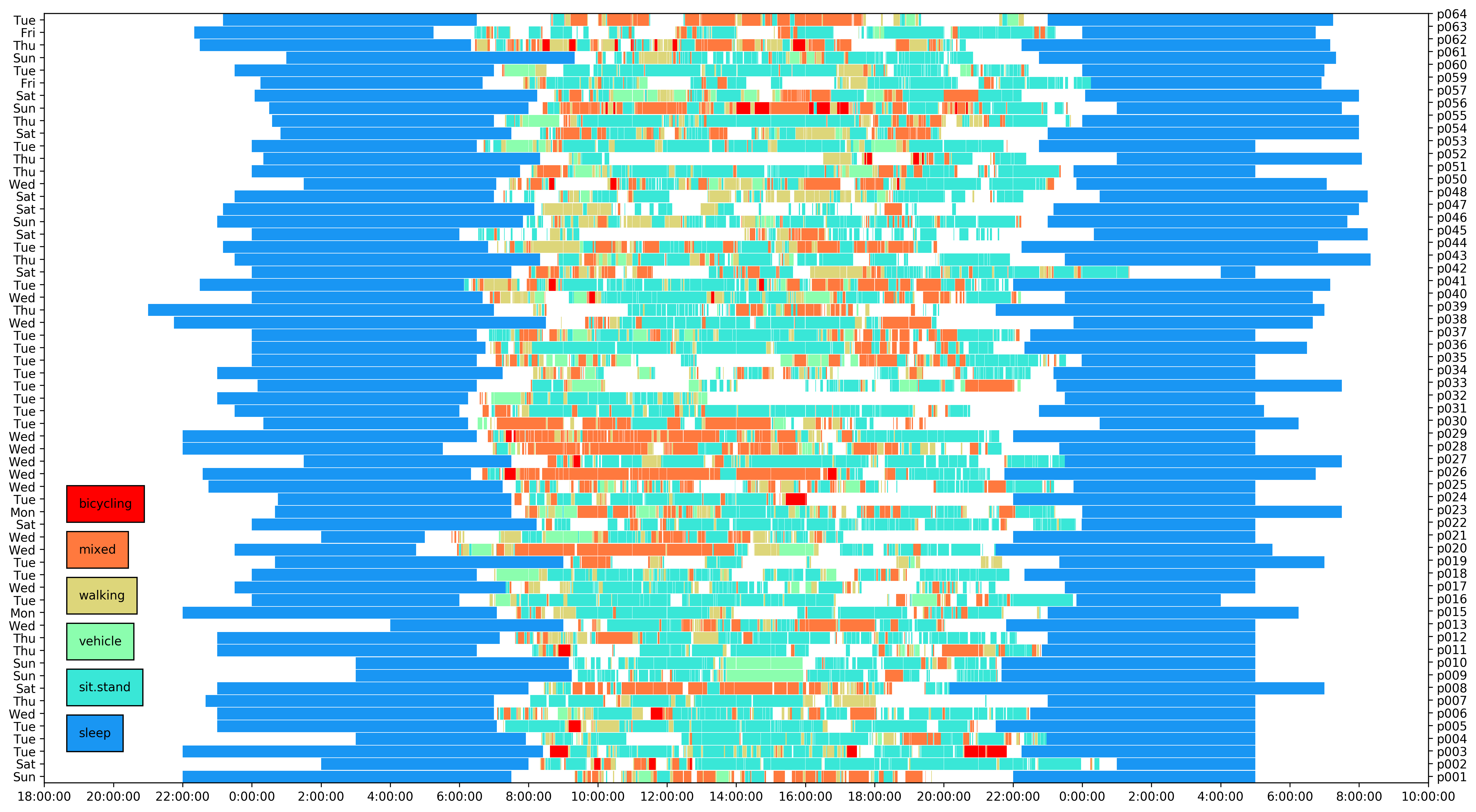


### **Fig. S7.** Diagram of a Hidden Markov Model. The sequence of discrete states $\left\{ \ldots,z_{t-1},z_{t},z_{t+1},z_{t+2},\ldots\right\}$ form a Markov chain. At each time step an observation is obtained by a draw from a probability distribution that is conditional on the value of $z$ at that time. This results in a sequence of observations $\left\{ \ldots,y_{t-1},y_{t},y_{t+1},y_{t+2},\ldots\right\}$.

### **Appendix S1.** Self-completed touchscreen questions used to determine participant characteristics in this study: the UK Biobank study 2013-2015. Our categorisations are included after the ‘->’ symbol.

MorningEveningPerson :

Do you consider yourself to be?

- Definitely a ‘morning’ person -> morning
- More a ‘morning’ than ‘evening’ person -> morning
- More an ‘evening’ than a ‘morning’ person -> evening
- Definitely an ‘evening’ person -> evening
- Do not know -> none
- Prefer not to answer -> none

Workers-cycle & workers-carUser:

What types of transport do you use to get to and from work?

(you can select more than one answer)

- Car/motor vehicle (checked -> worker-carUser)
- Walk
- Public transport
- Cycle (checked -> worker-cycle)
- None of the above
- Prefer not to answer

JobActivity:

Does your work involve heavy manual or physical work?

- Never/rarely = no
- Sometimes = no
- Usually = yes
- Always = yes
- Do not know = NA
- Prefer not to answer = NA

Working:

Which of the following describes your current situation?

(You can select more than one answer)

- paid employment or self-employed -> working
- Retired -> retired
- Looking after home and/or family -> not working
- Unable to work because of sickness or disability -> not working
- Unemployed -> not working
- Doing unpaid or voluntary work -> not working
- Full or part-time student -> not working
- None of the above -> NA
- Prefer not to answer -> NA

Self-rated health:

In general how would you rate your overall health?

- Excellent -> Excellent
- Good -> Good
- Fair -> Fair/poor
- Poor -> Fair/poor
- Do not know -> NA
- Prefer not to answer -> NA

### **Appendix S2.** Definition of behaviour classes mapped original image annotation of codes from Compendium of Physical Activities codes.

**2a)** Activity prediction (6-class):

| Class | Physical Activity Compendium Codes |
| --- | --- |
| bicycling | 1010, 2010, 2019 |
| mixed | 2048, 2050, 2060, 2065, 2070, 2115, 2120, 3010, 5010, 5020, 5025, 5032, 5035, 5041, 5051, 5053, 5060, 5070, 5080, 5090, 5092, 5095, 5100, 5121, 5131, 5140, 5146, 5148, 5171, 5175, 5181, 5183, 5185, 5186, 5192, 5197, 6124, 6126, 8025, 8050, 8095, 8135, 8192, 8230, 8245, 9015, 9085, 9101, 10074, 11050, 11115, 11192, 11413, 11475, 11540, 11600, 11615, 11795, 11870, 12150, 13000, 13009, 13020, 13040, 15090, 15235, 15680, 15690, 17031, 18070, 20039, 21017, 21070 |
| sleep | 7030 |
| sit.stand | 5060, 5080, 5146, 5160, 5170, 5185, 5190, 7010, 7021, 7040, 9015, 9020, 9030, 9045, 9050, 9055, 9060, 9065, 9070, 9071, 9075, 9100, 10074, 11580, 11585, 11600, 13009, 13030, 13035, 13036, 13045, 13046, 20000, 20005, 20030, 21000, 21005, 21010, 21016 |
| vehicle | 8055, 16010, 16015, 16016 |
| walking | 5035, 5060, 5147, 5165, 11791, 17070, 17082, 17133, 17150, 17161, 17165, 17250, 17270, 21070 |

**2b)** Energy expenditure prediction (11-class):

| Class | Physical Activity Compendium Codes |
| --- | --- |
| bicycling | 1010, 2019 |
| gym | 2010, 2060, 3010, 11192, 11540, 15680 |
| running | 12150 |
| sitting | 5190, 7010, 7021 |
| sitstand+activity | 5020, 5025, 5032, 5035, 5041, 5070, 5090, 5092, 5100, 5146, 5160, 5170, 5171, 5183, 5185, 5186, 5197, 6124, 6126, 8050, 8135, 8192, 8230, 9020, 9050, 9065, 9070, 9071, 9075, 9085, 9100, 9101, 10074, 11115, 11475, 11600, 11615, 13000, 13009, 13020, 13030, 13035, 13040, 13045, 20005, 20030, 21016, 21017 |
| sitstand+lowactivity | 5080, 9015, 9030, 9045, 9055, 9060, 11580, 11585, 13036, 13046, 20000, 21000, 21005, 21010] |
| sleep | 7030 |
| sports | 2048, 2050, 2065, 2070, 2115, 2120, 5175, 15090, 15235, 15690, 18070 |
| standing | 7040 |
| vehicle | 8055, 16010, 16015, 16016 |
| walking | 17070, 17082, 17133, 17150, 17161, 17165, 17250, 17270 |
| walking+activity | 5010, 5051, 5053, 5060, 5095, 5121, 5131, 5140, 5147, 5148, 5165, 5181, 5192, 8025, 8095, 8245, 11413, 11791, 11795, 11870, 17031, 20039, 21070 |

**Appendix S3.** Classification and regression trees in random forests.

For activity classification we use random forests which offer a powerful nonparametric discriminative method for multi-class classification that offers state-of-the-art performance. Predictions of a random forest are an aggregate of individual CART trees (Classification And Regression Trees). CART trees are binary trees consisting of split nodes and terminal leaf nodes. In our case, each tree is constructed from a training set of feature data along with ground truth activity classes. For a standard random forest, to train a tree from $N$ data points with $F$ features, we first select $N$ data points with replacement and $\sqrt{F}$ feature variables (without replacement), then carry out the CART algorithm as follows:

At each node split the training data for that node according to the threshold of one of the available features. We wish to choose which feature and which threshold by greedily optimising the purity of that split as measured by the total Gini impurity $I_{G}$. $I_{G}$ is a function over the classes of the data at that node $D$ and the data given to each of the two outgoing nodes, left $D_{L}$ and right $D_{R}$ as a result of the application of the threshold:

$$I_{G}\left( D,D_{L},D_{R} \right)=G\left( D \right)-\sum_{i\in\left\{ L,R \right\}} \frac{\left| D_{i} \right|}{\left| D \right|}G(D_{i})$$

Where the Gini impurity for one set of data $G\left( X \right)=1-\sum_{j=1}^{k} {P(c_{j}|X)}^{2}$ and $P\left( c_{j} | X \right)$ is the probability of a randomly-drawn data point being in a given class $c_{j}$ out of $k$ total classes. Thus $I_{G}$ is the cardinality-normalised sum of $G\left( X \right)$ over $D$, $D_{L}$ and $D_{R}$. A minimum is obtained when $D_{L}$ and $D_{R}$ are each composed of data of only a single class.

When choosing the threshold, we try every available feature with a range of possible threshold values over incoming data $D$ at that node. The optimum combination of feature and threshold becomes the decision process at that node. Thus the tree divides the feature space by axis-parallel splits. Repeat this process of creating binary splits at each node until the nodes have data points of only one class entering from its parent. This terminal node is now associated with that class.
