# Supplementary material for: Statistical machine learning of sleep and physical activity phenotypes from sensor data in 96,220 UK Biobank participants
Source: Sci Rep. 2018 May 21;8:7961. doi: 10.1038/s41598-018-26174-1 (PMC5962537; doi:10.1038/s41598-018-26174-1)
Supplement: Supplementary file 2 — Supplementary document [file 41598_2018_26174_MOESM2_ESM.docx]

**Statistical machine learning of sleep and physical activity phenotypes from sensor data in 96,220 UK Biobank participants.**

**Matthew Willetts, Sven Hollowell, Louis Aslett, Chris Holmes, Aiden Doherty**

# Supplementary Document

**Document S1.** Wearable Camera Annotation Training Protocol

Wearable cameras such as the SenseCam can take pictures at fixed intervals or in response to a change in a wearer’s environment. Their images provide a rich source of data for researchers interested in physical activities and the corresponding energy expenditures. In order to analyse the data, images must first be split and annotated for the activities of interests. We have designed a Wearable Camera Browser for this purpose (see https://github.com/activityMonitoring/wearable-camera-desktop-browser). This browser enables users to group images into albums that can be annotated with descriptive items. Annotated wearable camera data can then be extracted from the browser’s database and used for analysis.

In an effort to ensure reliability and efficiency in this annotation process, this training protocol attempts to provide training information for the ethics, annotation guideline, quality checks and data security. Researchers who plan to use this protocol to create annotated wearable camera data should first be trained. This protocol, developed by Aiden Doherty, Wong Tsz Yan, Nicole Gray, Lindsay Lee, and Salma Haque serves as a reference for research in the future. Comments for improving the protocol are highly appreciated.

# Training overview

There are five steps for researchers to complete prior to any annotation work:

1. undertaking the ethics training for handling image data (including data security practices);
2. learning to use the image browser software;
3. undertaking the annotation training; and
4. performing quality checks using Inter-Rater Agreement test (IRR test).

# Ethics training

Researchers wishing to use image-based data should be aware of related ethical issues. Albeit it is beyond the scope of this protocol to present a detailed account of the ethical issues that arise when images are viewed by a large team of researchers, the decision making of most research ethics boards (e.g. institutional review boards) are based on three core principles. These are

1. respect’s autonomy (the right of others to make their own informed decision);
2. non-maleficence (not doing harm, avoiding personal risks etc.); and
3. beneficence (doing good or promoting well-being); and
4. justice (ensuring that the benefits are equitable, including across those who are invited to participate vs. those who are not).

At a minimum standard, researchers who wish to annotate wearable camera images should complete a tutorial on research ethics. Study-specific ethics training, which lists out the necessary reading, relevant research ethics courses and important notes, is recommended.

All researchers must read the two declarations shown below which provide a clear list of journal articles related to research ethics of the wearable camera studies, a link to an online certificated course regarding protecting human research participants, and items of the important ethical considerations.

## Data security practices

All annotated data are stored on the local machine and need to be backed up periodically to prevent extensive data loss in the event that the machine is damaged. We recommend backing up the camera annotations regularly on a secure server. We also recommend making an archive copy of all participant data that will not be used for annotation.

**Ethical declaration for researchers viewing and annotating wearable camera images**

November 2016

**Study Name:**……………………………………………………………………

**Principal Investigator:** ……………………………………………………………

**Data Set:** ………………………………………………………………

Please initial box

| I confirm I have read and understood the ethical framework for wearable camera research:  Kelly, P., Marshall, S.J., Badland, H., Kerr, J., Oliver, M., Doherty, A.R., Foster, C. (2013) An Ethical Framework for automated-wearable cameras in health behaviour research. *American Journal of Preventive Medicine* 44(3); 314-319 |  |
| --- | --- |
| I confirm I have read and understood parts A & B of “Guidelines for Ethical Visual Research Methods”:  Cox, S. Drew, S. Guillemin, M. Howell, C. Warr, D. and Waycott, J. (2014) Guidelines for Ethical Visual Research Methods, The University of Melbourne, Melbourne. |  |
| I confirm I have read and understood ethical considerations of using wearable cameras to influence health behaviours:  Doherty, A.R., Williamson, W., Hillsdon, M., Hodges, S., Foster, C., Kelly, P. (2013) "Influencing health-related behaviour with wearable cameras: strategies & ethical considerations". Proceedings of SenseCam and Pervasive Imaging Conference, San Diego, USA, November 18-19, 2013 |  |
| I confirm I have completed Ethical Training appropriate to my institution.  Researchers from the University of Oxford can complete the course "Research Integrity: Social and Behavioural Sciences" through Weblearn: [https://weblearn.ox.ac.uk/portal/hierarchy/grad](https://weblearn.ox.ac.uk/portal/hierarchy/grad)    Alternatively, researchers can complete the following open access course:  “Protecting Human Research Participants” (NIH Office of Extramural Research”) - <https://phrp.nihtraining.com/> |  |
| I will undertake to protect the privacy and confidentiality of all participants and their wearable camera data |  |
| I understand that I must not disclose the content of any images to anyone who has not signed this agreement |  |
| I understand that I cannot make any copies of any images, email images or post copies to any internet sites (Facebook, Twitter, etc) |  |
| I understand that I must not send images over e-mail to any third-party vendor |  |
| I understand that any images used in presentations, papers or reports must be approved by the Study Principal Investigator before use |  |
| I understand that I must report images that depict illegal activities (including but not limited to abuse, violence or drug taking) to the Study Principal Investigator |  |

Name of researcher ……………………… Signature…………………… Date……………..

Principal investigator……………………… Signature…………………… Date………………...

# Browser training

Prior to learning the annotation work, the researchers should download the Doherty Wearable Camera Browser onto their computer and understand the operation. In the training, the researcher should know:

1. setting up the browser;
2. downloading participant images from either PC database or the Camera directly;
3. importing annotation list, choosing the date of interests, splitting and annotating images;
4. making amendment of image annotation; and
5. exporting annotated file in the CSV form (depends on the statistical analyses required).

Allowing public access to the browser and instructions, *Documentation of the Researcher Wearable Camera User Guide* is an open-course material available at <https://github.com/activityMonitoring/wearable-camera-desktop-browser/wiki>. Text description and video tutorials are accessible with links in the user guide. For detailed information, please visit the website.

# Annotation training

In this protocol, the researcher will find how “events” (i.e. a collection of images) are split and annotated from one of the five categories: uncodeable, occupation, transport, home activity and leisure. Sub-categories are listed with supplementary notes.

Each item is linked with specified Metabolic EquivalenT (MET) value. MET values can be referred to the latest version of Compendium of physical activities published in 2011.^[[1]](#endnote-1)^ This Compendium supplies a direct translation of the mass-specific energy expenditure of activities.

Development of this annotation taxonomy is an on-going process and any comments and recommendations are hugely appreciated.

**Extracting physical activity information from wearable camera images: annotation protocol**

## Events

An “event” is a series of images grouped thematically. Firstly, the manual will run through how to split events accurately.

## Three-image rule

Activities will be split into episodic “events”, each containing at least three images. The start of an event is the first image in a set of three (or more) consecutive images that depict the same activity, or where the researcher is almost certain that the same activity is occurring across the images. An event ends when the next event begins, so activities that last less than three images will be grouped with the preceding event.

## Dominating activities

It is possible that the event does not depict the same activity across all images. Accordingly, the researcher should annotate the dominating behaviour, in which the largest proportion of the event depicts. Researchers should prefer the specific annotation items to generic counterparts, unless the primary activity does not match with any specific annotation item. Figure 1 illustrates how events should be split and which behaviour should be annotated later.

When the dominating behaviour is in doubt, the researcher should annotate the event with a more generic description. For example, when a series of images show that the participant shut the window, took the key and locked the door as he/she was ready to leave the home; there is no dominating event or specific annotation for such household tasks. In this case, “home activity;miscellaneous;5165 (generic) walking non-cleaning task such as closing windows lock door putting away items” should be annotated.

**Figure 1. An example of events split.**


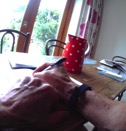

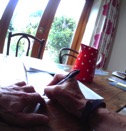

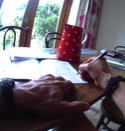

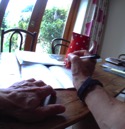

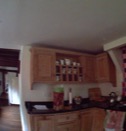

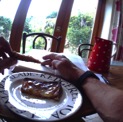

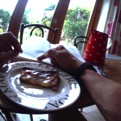

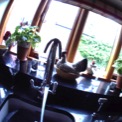

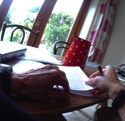

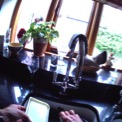

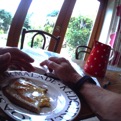

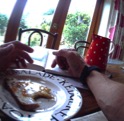

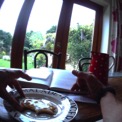

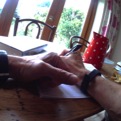

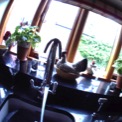


**Split events here**

**O = office/computer work general (11580) K= kitchen activity (5035) E= eating sitting (13030)**

**1) Images: OOOKOEOKOK EEEER**

**2) Split image: OOOKOEOKOK EEEER** The first event, whilst consisting of a variety of actions, is annotated as ‘office/computer work general’ as this is the dominant activity. Then the events are split at the red text as this is when we find 3 or more consecutive images depicting the same behavioural activities.

**Event 1- Office Work**

**Event 2- Eating**

## Primary activities

As a participant might do multiple tasks at the same time, the researcher should identify the primary activity for the annotation. The primary activity is the one which the participant was actively performing. Watching the movements of the participant’s hands would help with this identification. In image 1, the participant was looking at his laptop showing a video. In this case, the researcher should annotate the primary activity as desk work, rather than sitting watching TV, despite the TV being on in the background.


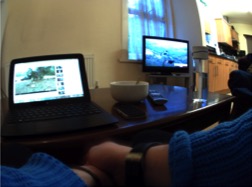


**Image 1. An example of “home activity; walking/standing/sitting;9030 desk entertainment/hobby (with or without eating at the same time)”.**

## Image annotation

After uploading photos and the import annotation list, the researcher should annotate events beginning with the lowest tier in the list. Table 1 summarises the general rules of the annotation list for looking for the appropriate categories.

| **Categories** | **Rules** |
| --- | --- |
| Uncodeable | When the images are blocked and/or dark, or the images show that the participant took off the camera. |
| Occupation | The events after the participants had entered the working place and before they left that. |
| Transportation | The events after the participants had left their destination (e.g. homes, working place, churches, restaurants, shopping malls) for a certain activity and before they entered into another one. |
| Home activity | The events taken place at the participants’ home (guest’s house is regarded as a leisure place). |
| Leisure | The events that did not fall into the above categories. |

**Table 1. A summary of the general rules for identifying events under each category**

The next section is the descriptions of sub-categories under these major categories. Supplementary notes are provided for a better understanding of appropriate annotation.

**Uncodeable**

| **Lowest tier items** |
| --- |
| Camera taken off * (MET value: undefinable) |
| Image dark**/blurred/obscured (including camera being blocked) (MET value: undefinable) |

**Table 2. Categorisation of uncodeable images.**

*A series of images in which the position of the photo remains the same in relation to fixed objects. Lighting commonly changes without movement of the images. There is an exceptional case for this annotation: if the researcher can clearly identify the person captured in the images to be the participant per se over the course when the camera was taken off, the researcher should annotate the observed events that the participant was doing. Image 2 shows the image before the camera was taken off. The sleeve of the participant was red in colour. In the subsequent images in image 3 the camera was taken off, but the person in the images was in fact the participant. Therefore the corresponding annotation should be “home activity;miscellaneous;sitting;9030 sitting desk entertainment/hobby (with or without eating at the same time)”.


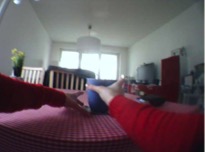


**Image 2. An image before the camera was taken off.**


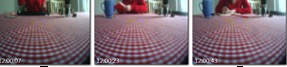

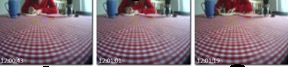


**Image 3. A series of images over the period when the camera was taken off.**

****Dark image rule**

To determine the annotation of darkened images, a rule of thumb is that a researcher cannot be certain about what is happening out of context (i.e. when looking at those images separately without referencing the previous and the following events).

### **Occupation**

| **Sub-category 1**^[[2]](#endnote-2),^^[[3]](#endnote-3)^ | **Sub-category 2** |
| --- | --- |
| Agriculture/forestry/fishing |  |
| Construction |  |
| Installation/maintenance/repair |  |
| Manufacturing |  |
| Office and administrative support |  |
| Personal services |  |
| Public admin/education/health | Public admin and defence  Education*  Health  Sports |
| Transportation and material moving |  |
| Miscellaneous |  |
| Interruption** | Sitting  Standing  Walking  Miscellaneous |

**Table 3. Categorisation of occupation.**

***Sitting in lectures/ seminars/ talks**

The annotation of “9065 students/attending seminars or talks, sitting in class including note-taking or class discussion” would be suitable for participants who were attending lectures, seminars alike. However, for students who were shown to be working on assignments in a library or in a study common area, the annotation should be “occupation; office and administrative support; 11580 office work/computer work general.”

****Interruption**

This includes items such as, walking on job in office, sitting in toilet, and walking downstairs.

### **Transportation**

| **Sub-category 1** | **Sub-Category 2** |
| --- | --- |
| Walking* |  |
| Private transportation |  |
| Public transportation |  |
| Waiting** |  |

**Table 4. Categorisation of transportation.**

Often participants will complete other tasks whilst undergoing a means of transport or whilst waiting, for example eating whilst riding in a car or truck, using the phone whilst standing or sitting waiting for the bus, and completing work whilst on a train. However transportation is always the dominant activity, and therefore the appropriate annotation should be selected from this category.

***Walking**

Researchers should identify whether walking was the single means to reach the destination or as a transitional transportation means. If it is the case that walking was the single means, the researcher also has to clarify whether it was for work or other purposes. For example, if the participant left home to a bus station as he took the bus to the workplace, the researcher should annotate the walking event as “transportation; walking; 17161 walking not as the single means of transport”.

In addition, over the course of walking, it is possible that the participant met others and stopped for a chat, or stopped to look at their smartphone. On these occasions, the researcher should annotate the event as “transportation; walking;9071 standing miscellaneous (talking to others etc.)”.

### **Home activity**

| **Sub-category 1** | **Sub-category 2** | **Sub-category 3** |
| --- | --- | --- |
| Child/elderly/pet care | Child care *  Elderly care  Pet care |  |
| Eating** |  |  |
| Home repair | Indoor  Outdoor |  |
| Household chores^[[4]](#endnote-4),^^[[5]](#endnote-5)**^ | Washing/ironing/mending clothes  Grocery shopping  House cleaning  Preparing meals/cooking/washing dishes |  |
| Lawn and gardening^[[6]](#endnote-6)^ | Gardening service  Lawn care service  Tree and shrub service |  |
| Leisure | Activities for maintenance of a household  Individual activities | With children  With animals  Miscellaneous*** |
| Self care*** |  |  |
| Miscellaneous | Sitting****  Standing  Walking***** |  |

**Table 5. Categorisation of home activity.**

The researcher should note that both the front and back garden of a household are included in the household category. Therefore, annotations such as “walking household without observable load” and “kitchen activity general cooking/washing/ dishes/ cleaning up” should be used when walking in the garden and when cooking a barbecue, respectively.

***Childcare standing occasional lifting**

This includes activities such as changing a nappy, when the participant was clearly standing and therefore had to lift the child.

****Eating**

Firstly, drinking only is still regarded as an eating event. Secondly, researchers have to pay attention to the presence and the number of people in the image. If the participant was eating alone or with someone in the images, the event should be annotated as “home activity; eating; 13030 eating sitting alone or with someone. If there were two or more people captured in the images, researchers should choose “home activity; leisure; activities for maintenance of a household;9100 social activities involving sitting relaxing talking eating with more than one person”. Finally, if the participant is walking whilst eating the appropriate walking annotation should be used, rather than an eating annotation.

*****Miscellaneous**

Of note is the annotation “home activity; leisure; activities for maintenance of a household; 9100 social activities involving sitting relaxing talking eating with more than one person”. It is used when there was more than one person eating or talking with the participant, sitting. For talking to a person sitting however, the corresponding annotation is “home activity; miscellaneous; sitting; 9055 sitting/lying talking in person/using a mobile phone/smartphone/tablet or talking on the phone/computer (skype chatting)”.

******Self care**

For activities typically performed in the bathroom, such as, grooming, washing hands, shaving, brushing teeth and putting on make-up, researchers should annotate the event as “home activity; self care;13040 (generic) self care such as grooming/washing hands/shaving/brushing teeth/putting on make-up not eliminating and bathing (not necessary in the toilet)”.

If the participant is measuring blood pressure/blood sugar levels from home then the annotation “home activity; self care; 13036 taking medication” should be used.

******Sitting**

The following table details the circumstance under which relevant events should be annotated.

| Items in the lowest tier | Descriptions |
| --- | --- |
| 5080 sitting non-desk work (with or without eating at the same time) | Desk must be absent; for work like sewing and knitting, choose the appropriate annotation under “home activity; household chores; washing/ironing/mending clothes”; and for art work, including wrapping a gift, choose the appropriate annotation under “home activity; leisure; individual activities”. For all work performed not on the desk, even for writing and using the laptop, researchers should opt for non-desk work, see image 4. |
| 7010 sitting/lying and watching television with TV on as the primary activity | TV must be on; watching TV should be the primary activities (not eating, looking at smartphone, reading book etc. at the same time). If the participant is standing and watching TV use the appropriate standing code. |
| 9030 sitting desk entertainment/hobby (with or without eating at the same time) | Desk must be present; the nature of work was entertaining, such as visiting social media and watching videos. For art and craft work, please choose the annotation under “home activity; leisure; individual activities”. If uncertain about the nature of the work choose this option not office work, see image 5. |
| 9045 sitting playing traditional video game computer game | The examples include sitting Wii games and NDS. |
| 9055 sitting/lying talking in person/using a mobile phone/smartphone/tablet or talking on the phone/computer (skype chatting) | This annotation should also be used when the participant is using a smart watch, and when the participant is reading to/ being read to by a child whilst sitting. The exception is when the participant was talking to multiple people, where the annotation would be “9100 social activities involving sitting relaxing talking eating with more than one person”. |
| 9060 sitting/lying reading or without observable/identifiable activities | Participant should be reading books, papers or newspaper (albeit not necessarily on the desk) or performing unidentifiable activities (i.e. not captured by images). However if participant was making notes as they read academic work, the researcher should identify whether note taking or reading was the dominant activity- if it was note taking the annotation should be “11580 office work such as writing and typing (with or without eating at the same time)” |
| 11580 office work such as writing and typing (with or without eating at the same time) | Desk must be present; paper work such as writing notes and letters, or computer work on spreadsheets, word documents, typing emails alike is shown. Filling in the diary of *the* *CAPTURE-24 study* on the desk is also regarded as office work. If researchers are uncertain about the nature of work, “desk work” should be preferred. See image 6. |

**Table 6. A summary of the descriptions of sitting activity.**


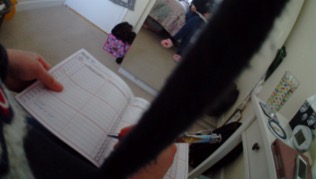


**Image 4. An example of “home activity;miscellaneous;sitting;5080 sitting non-desk work (with or without eating at the same time)”**


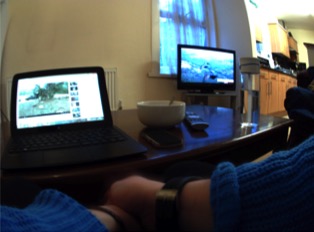


**Image 5. An example of “home activity;miscellaneous;sitting;9030 desk entertainment/hobby (with or without eating at the same time)”.**


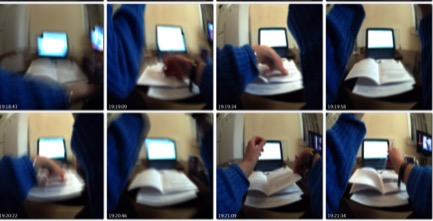


**Image 6. An example of “11580 office work such as writing and typing (with or without eating at the same time)”.**

### **Leisure**

| **Sub-category 1** | **Sub-category 2** | **Sub-category 3** |
| --- | --- | --- |
| Eating* |  |  |
| Sports | \| Ball games \|  \| \| --- \| --- \| \| Biking and racing \| Automobile traveling  Cycling \| \| Conditioning** \|  \| \| Fighting sports \|  \| \| Fishing and hunting \|  \| \| Gymnasium and athletics \| Gymnasium  Athletics \| \| Water games \|  \| \| Winter games \|  \| \| Miscellaneous \|  \| | |
| Dancing |  | |
| Music playing |  | |
| Recreation | Outdoor  Indoor | |
| Religious activities |  | |
| Miscellaneous | Sitting  Standing  Walking**** | |

**Table 7. Categorisation of leisure.**

***Eating**

Drinking only is also an eating event. Any takeaway foods, including ordering food in a canteen, should be annotated as “leisure; eating; 5060 buying foods or drinks as a takeaway”. When the participant eats the food they have purchased an alternative annotation can be used. As with home activities, eating with multiple people is annotated as “leisure; miscellaneous; sitting; 9100 social activities involving sitting relaxing talking eating with more than one person”.

****Warm-up or cool-down exercise**

For there is no specific MET score for warm-up or cool-down exercise the closest description of the stretching movements would be “leisure;sports;conditioning;2060 health club exercise.”

*****Generic walking and occasional standing (no more than two consecutive images)**

This includes activities such as walking around a farm or park, or participating in a walking tour. However, when the participant is standing for 3 or more images an alternative annotation to describe the activity should be used. Also, the annotation “leisure;miscellaneous;walking;5060 shopping miscellaneous” should be used for activities such as visiting the bank or the post office, due to the similar nature of the physical activity.

## Case studies for annotation

### **Case study 1. Preparing for riding a bike**

Usually, the participant would move their bike from the house to the road. Here, the researcher should annotate the event as “home activity; miscellaneous; walking; 5121 walking with moving and lifting loads such as bikes and furniture”. Subsequently, when the participant was on the bike the annotation changes to “transportation; private transportation; 1010 bicycling”. If the participant moved the bike in the office, or other places, “occupation;interruption;11795 walking on job and carrying light objects such as boxes or pushing trolleys” and “leisure;miscellaneous;walking;21070 (generic) walking and occasional standing (no more than two consecutive images)” should be chosen respectively.


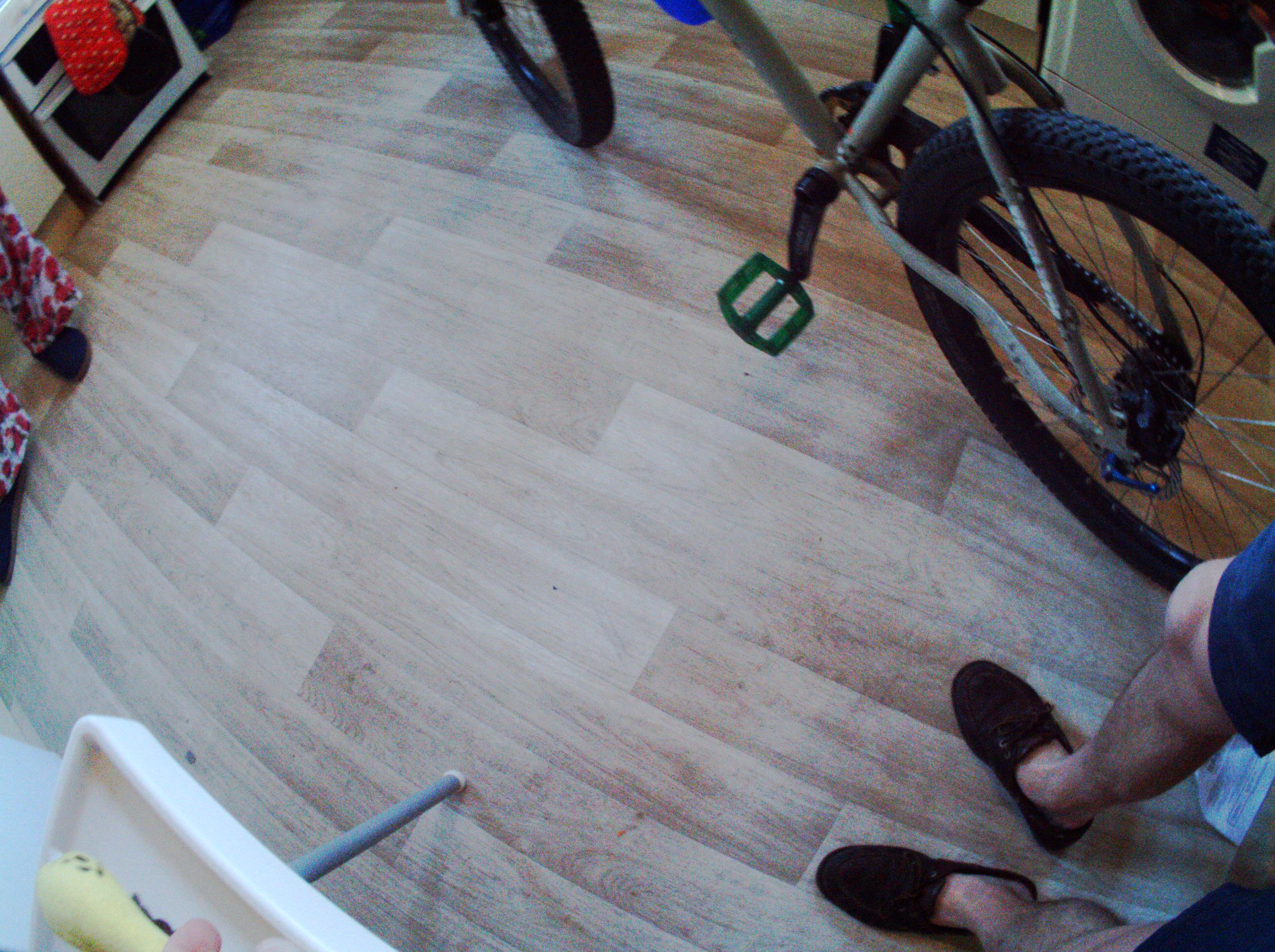

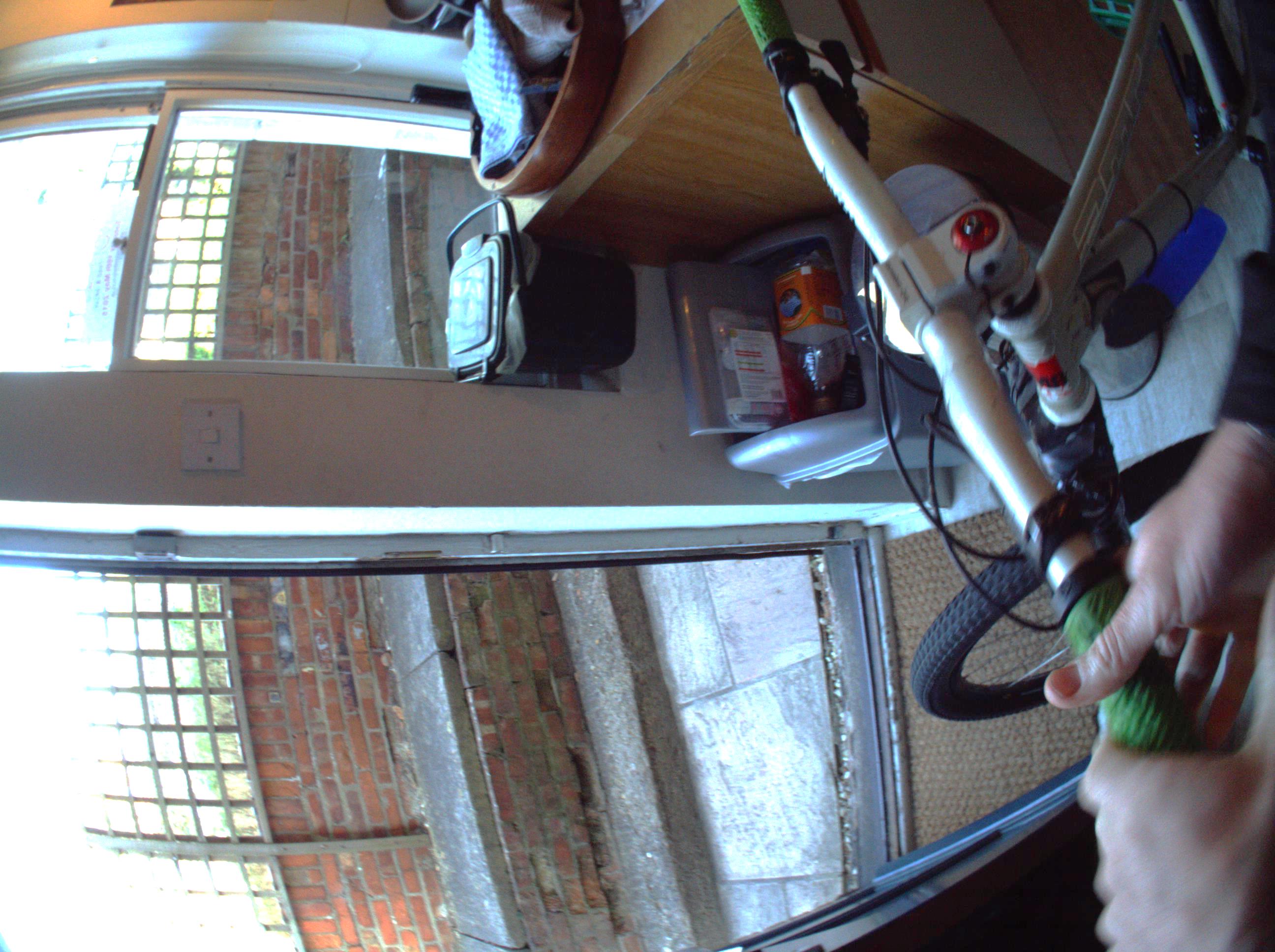

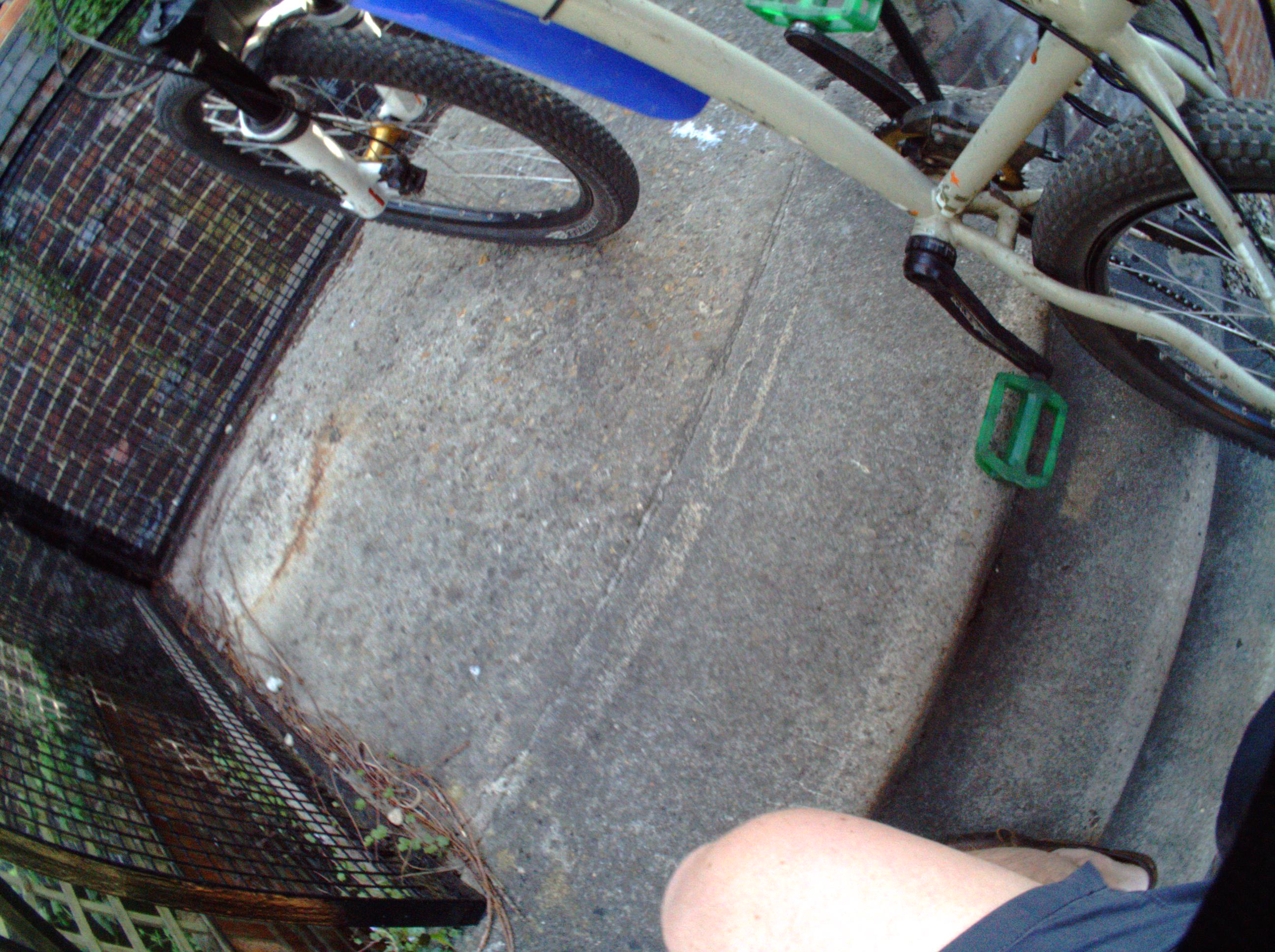

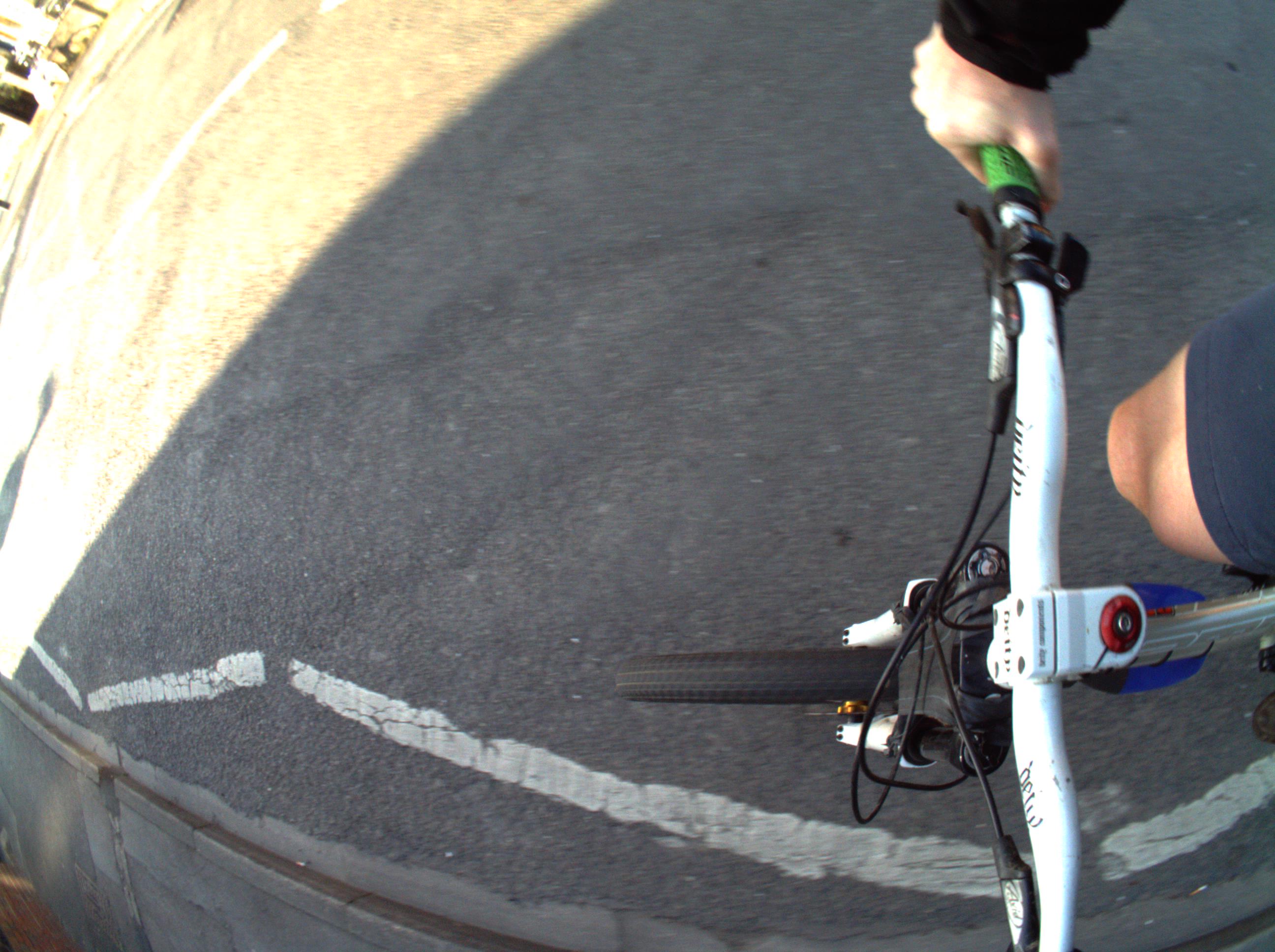

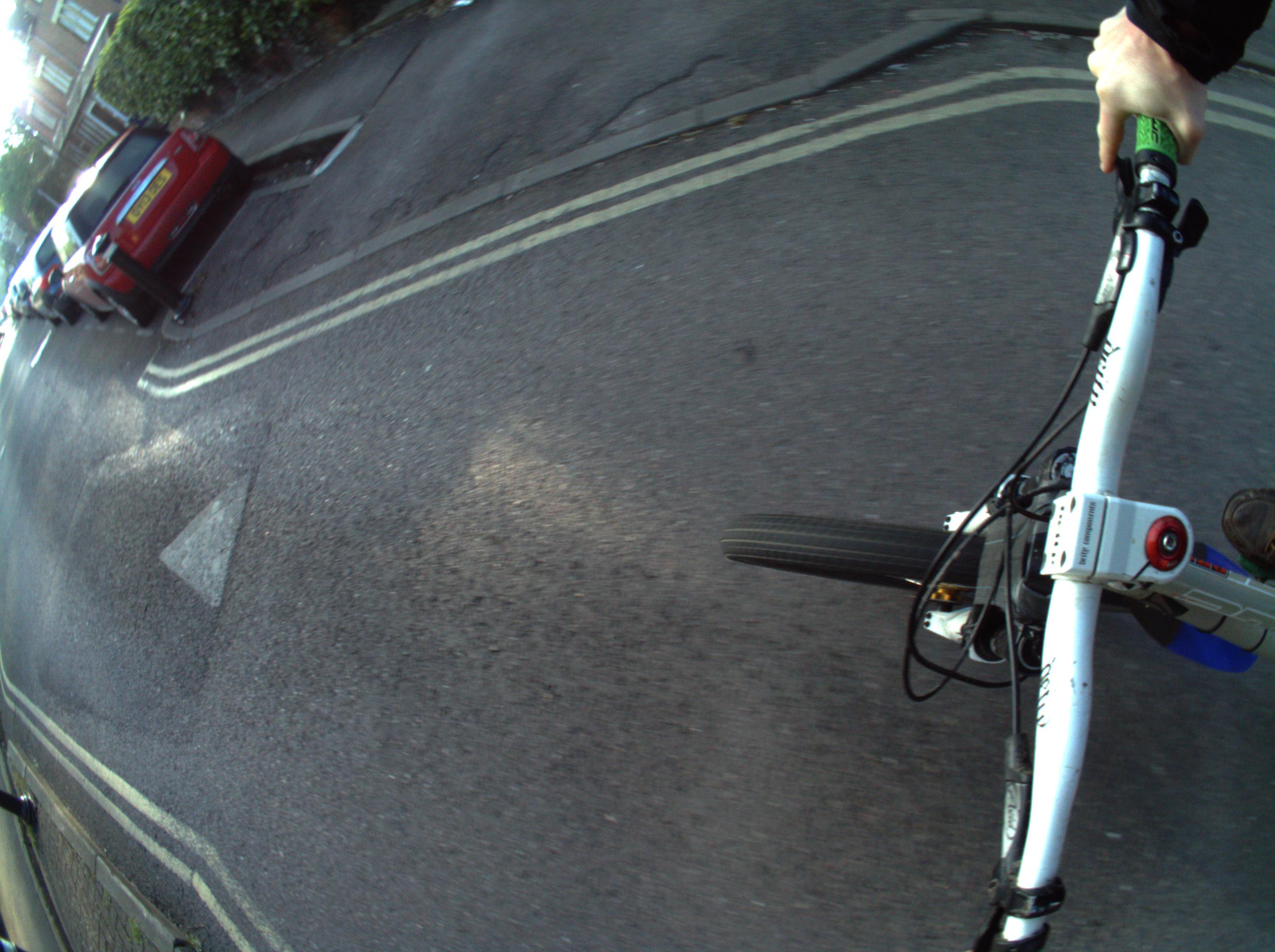

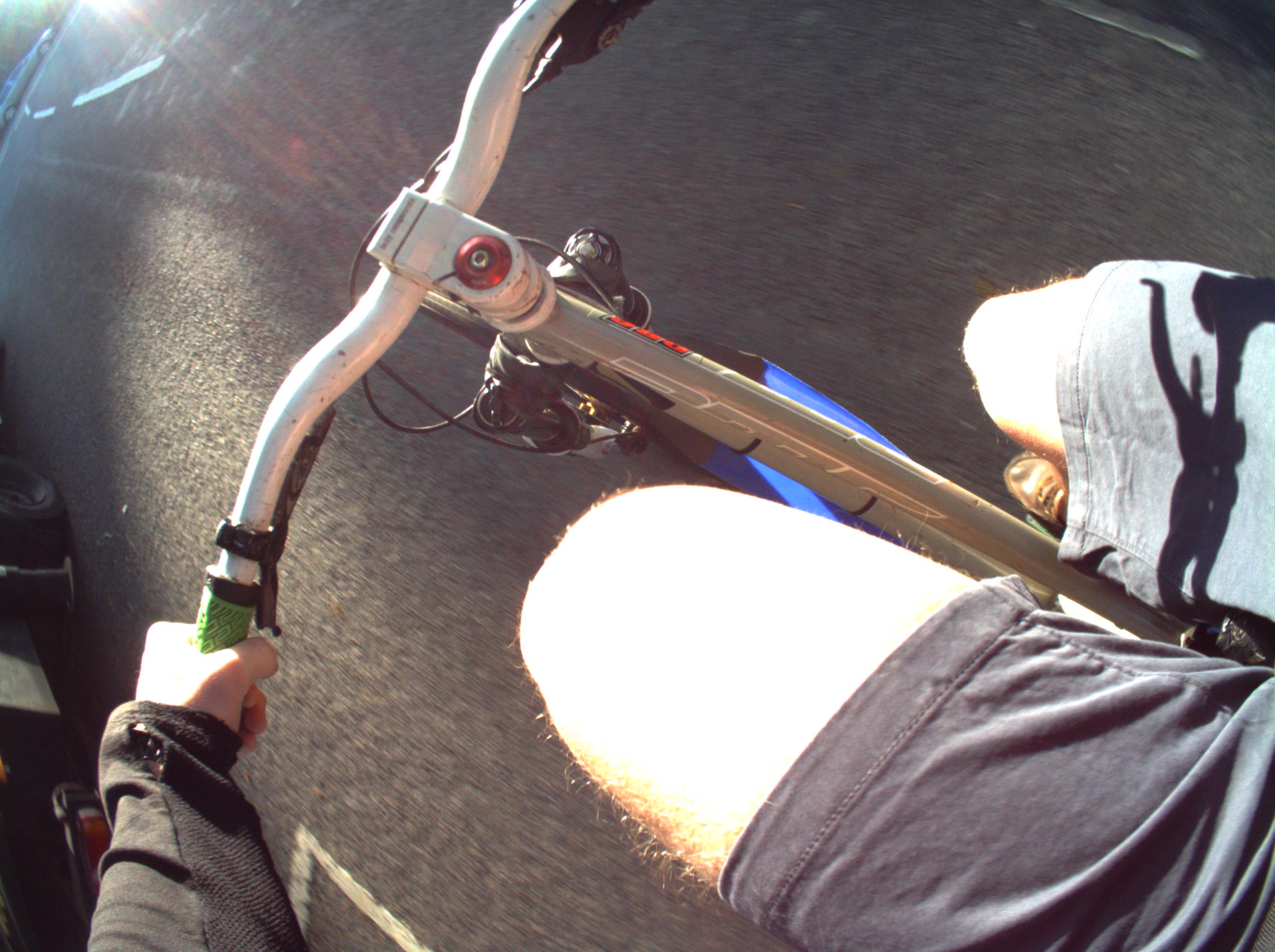

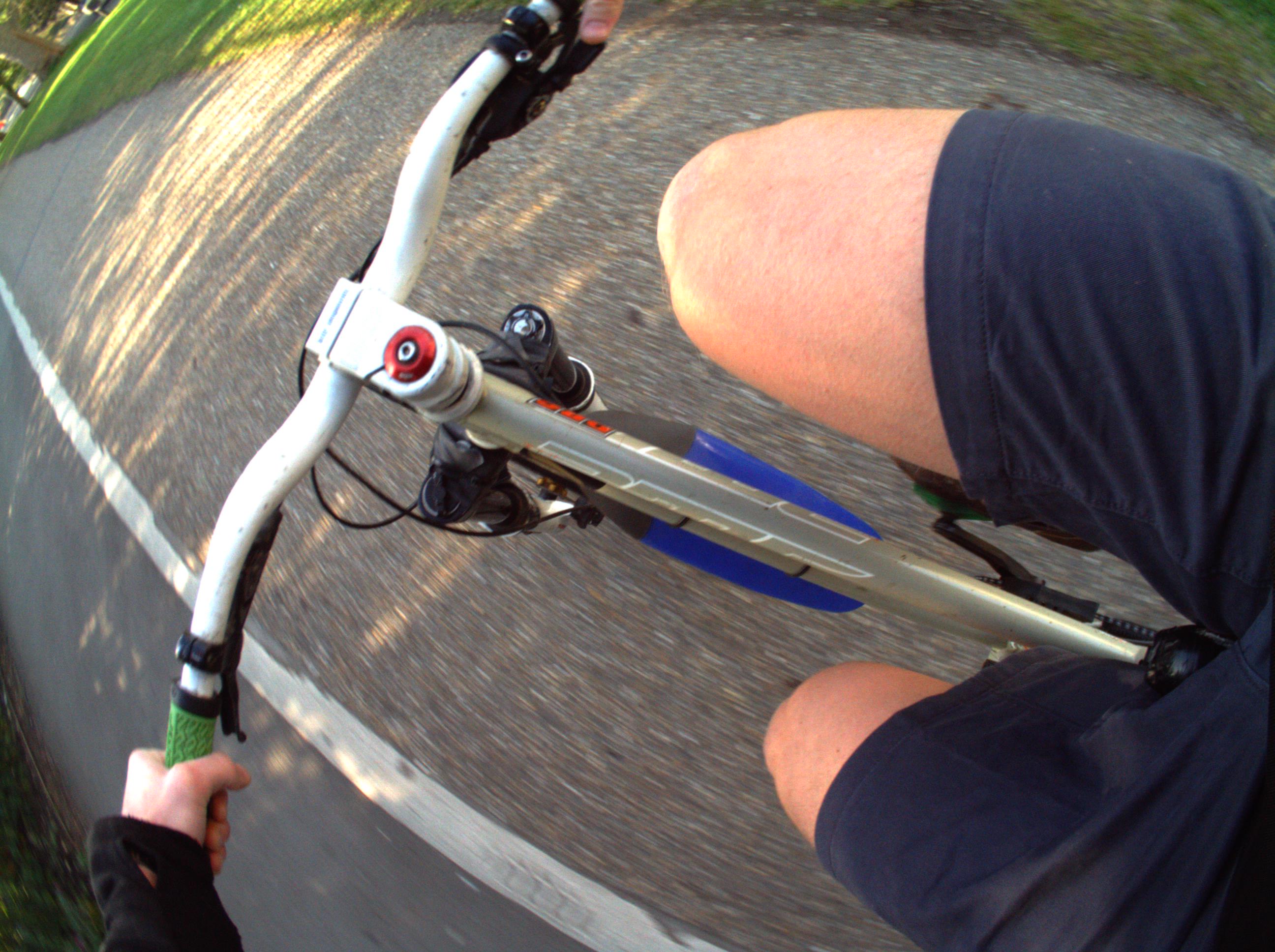

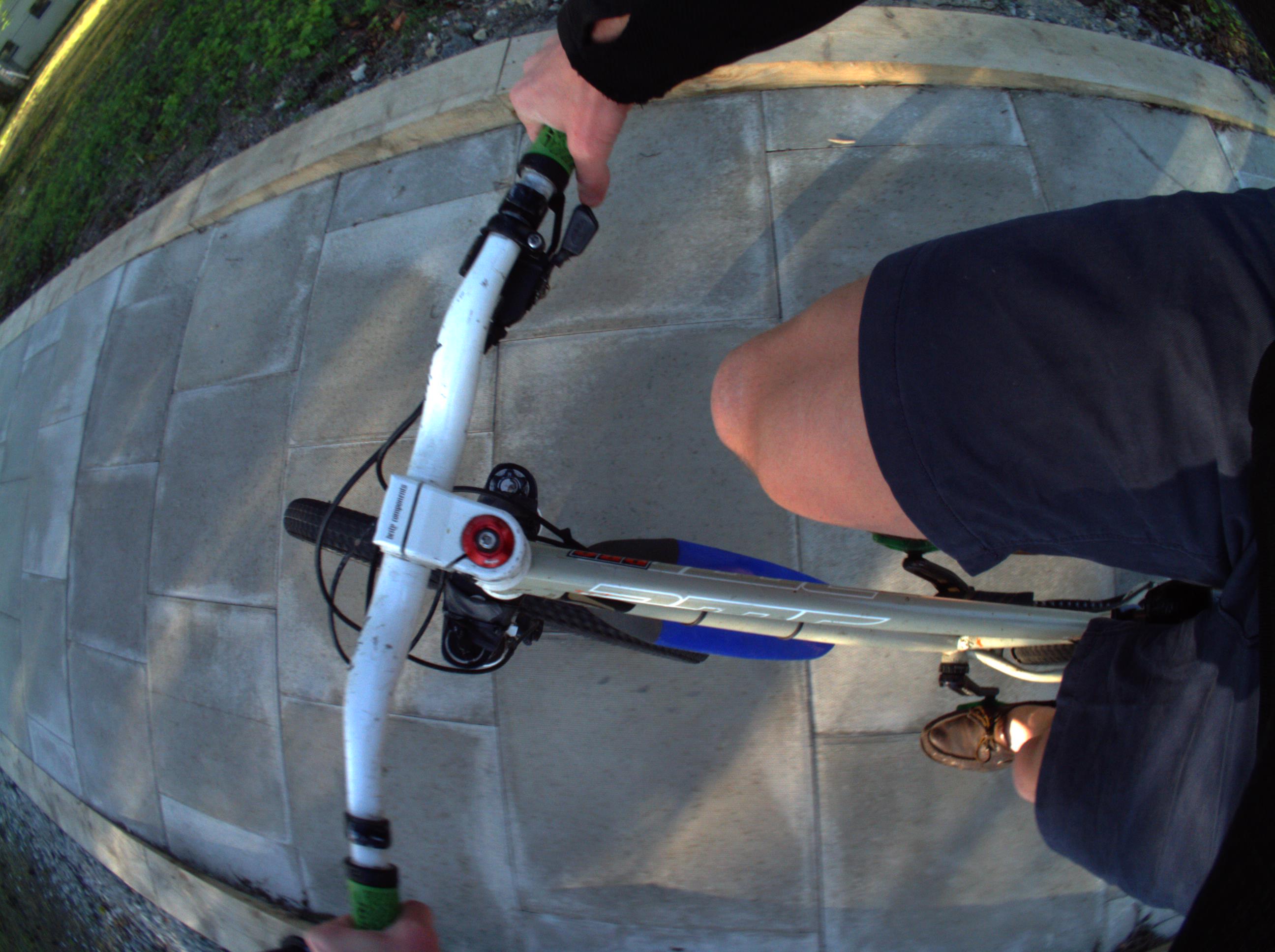


Here, the first 3 images would be annotated with “home activity; miscellaneous;walking;5121 walking with moving and lifting loads such as bikes and furniture”, and the remaining 5 images with “transportation; private transportation;1010 bicycling”. However, if the bicycling was not a means of transport and was for pleasure/exercise, the annotation “leisure; sports; biking and racing; 1010 bicycling for pleasure” should be used. This distinction can often be determined by reviewing the start location compared to the end destination.

### **Case study 2. Walking in office and walking as transportation means**


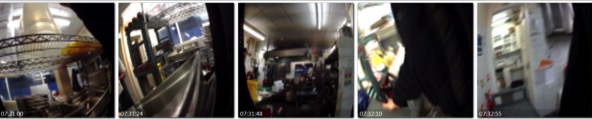


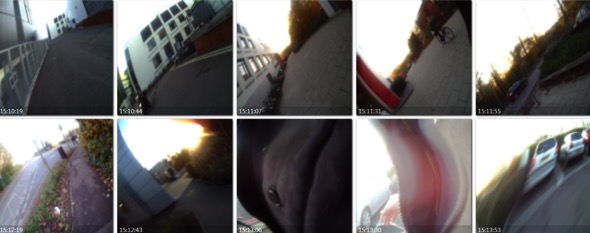


These are the series of images showing the participant leaving his office. According to the general rule, the first five images are grouped as an event of “occupation;interruption;11791 walking on job in office or lab area”, while the remaining ten are annotated under the main category of transportation, depending on whether the participant walked to his home on foot or by other transport means.

### **Case study 3. Sitting activities at home**


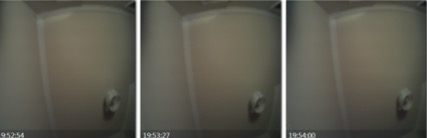


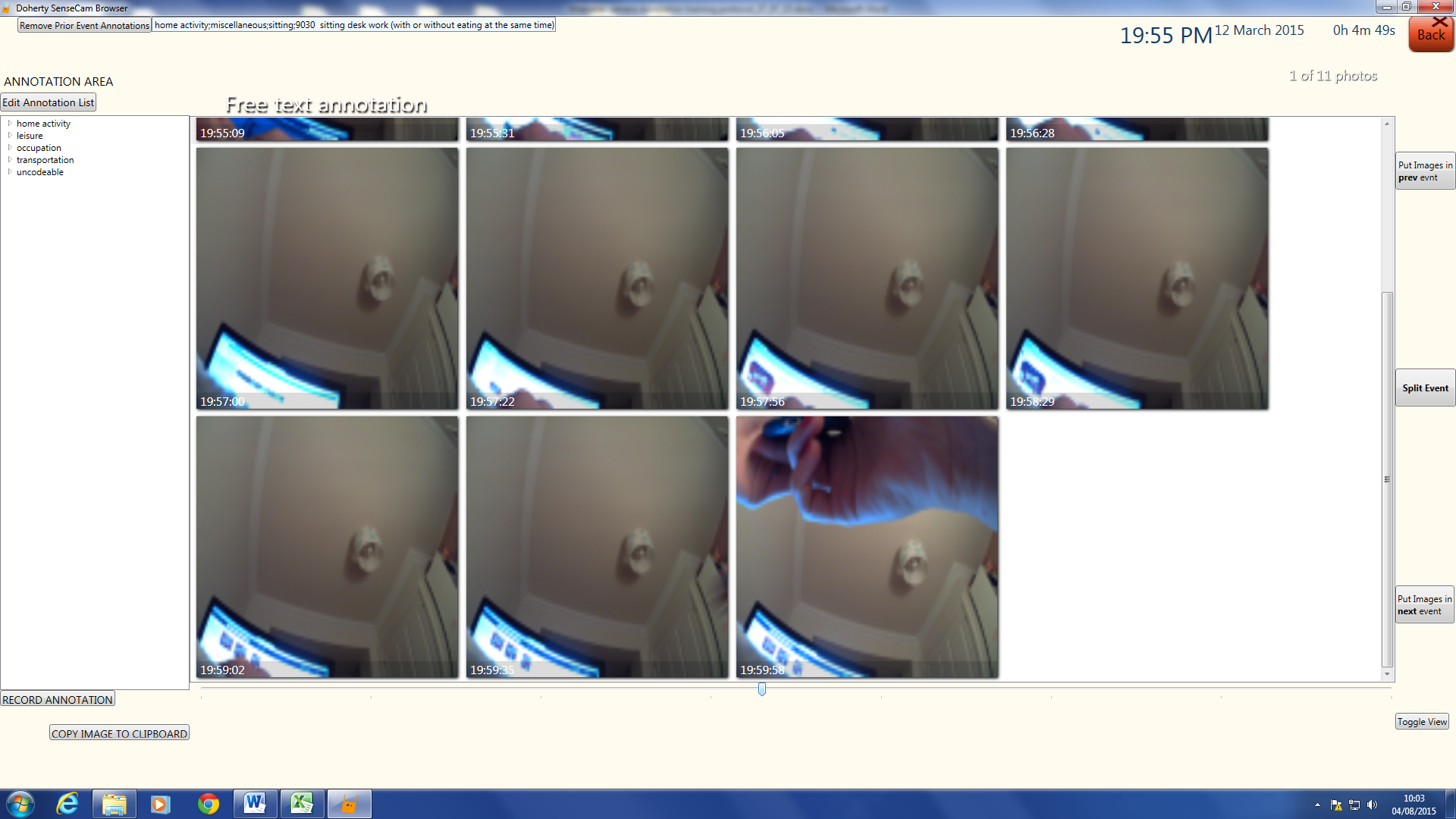

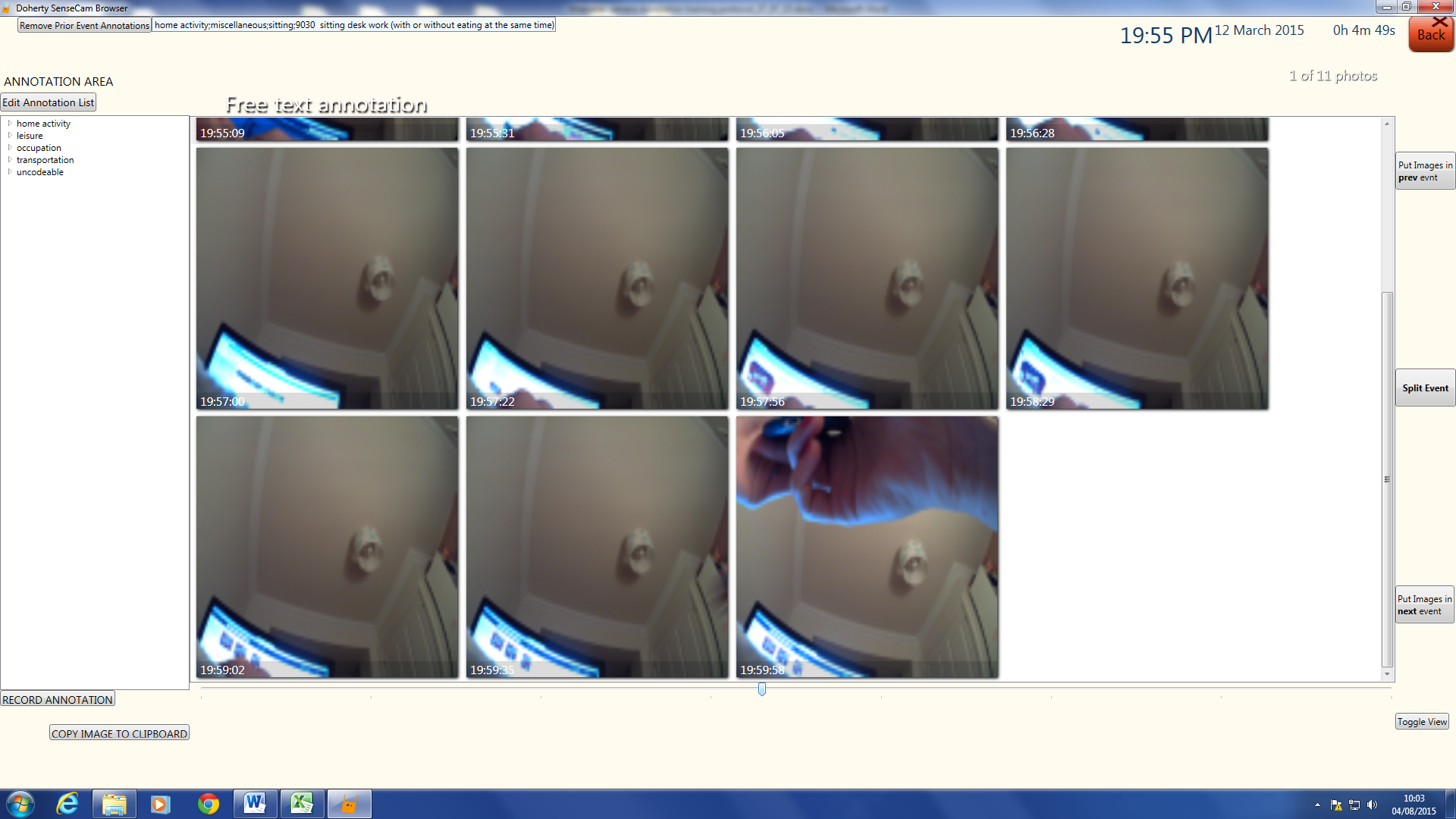

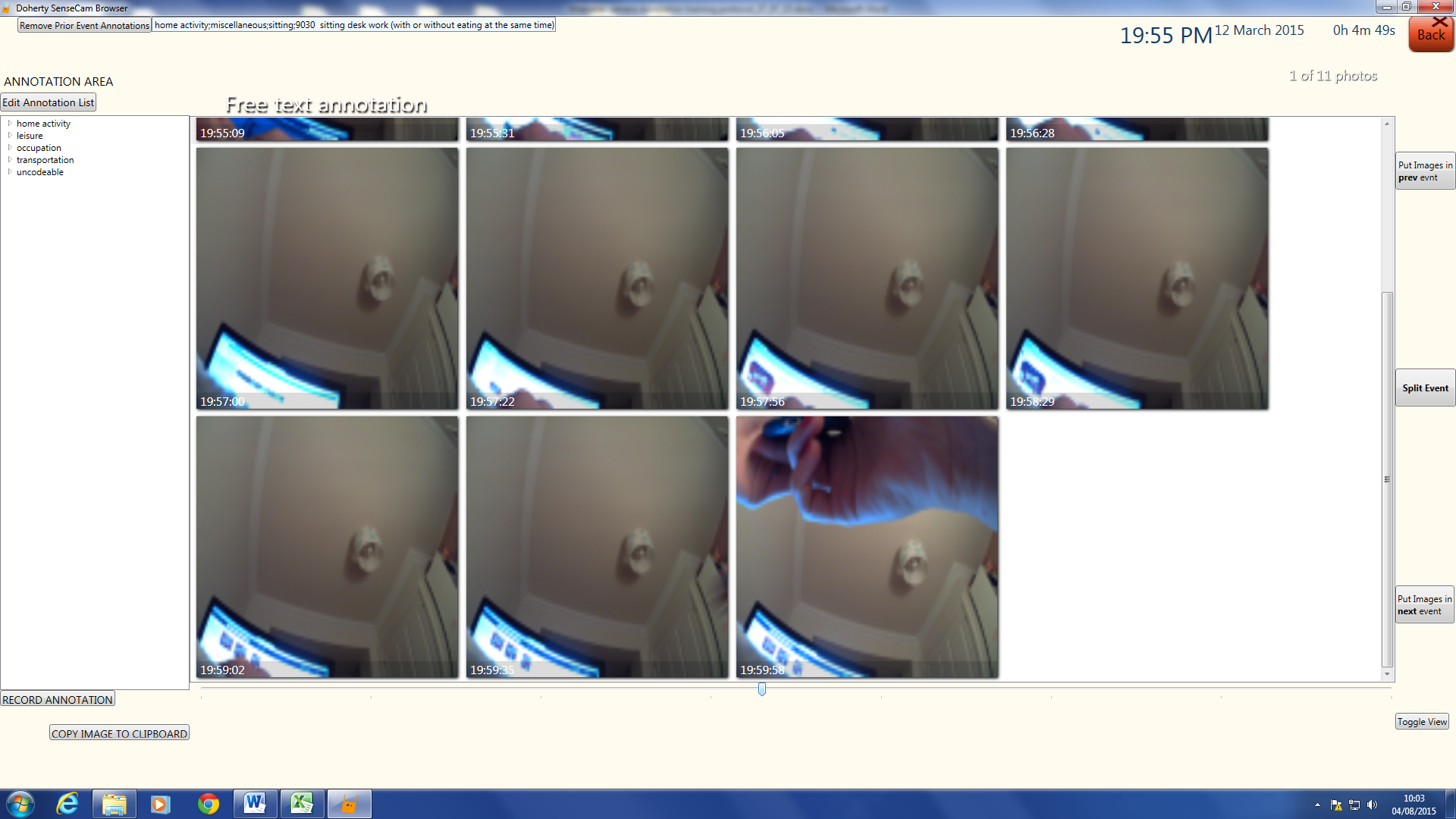

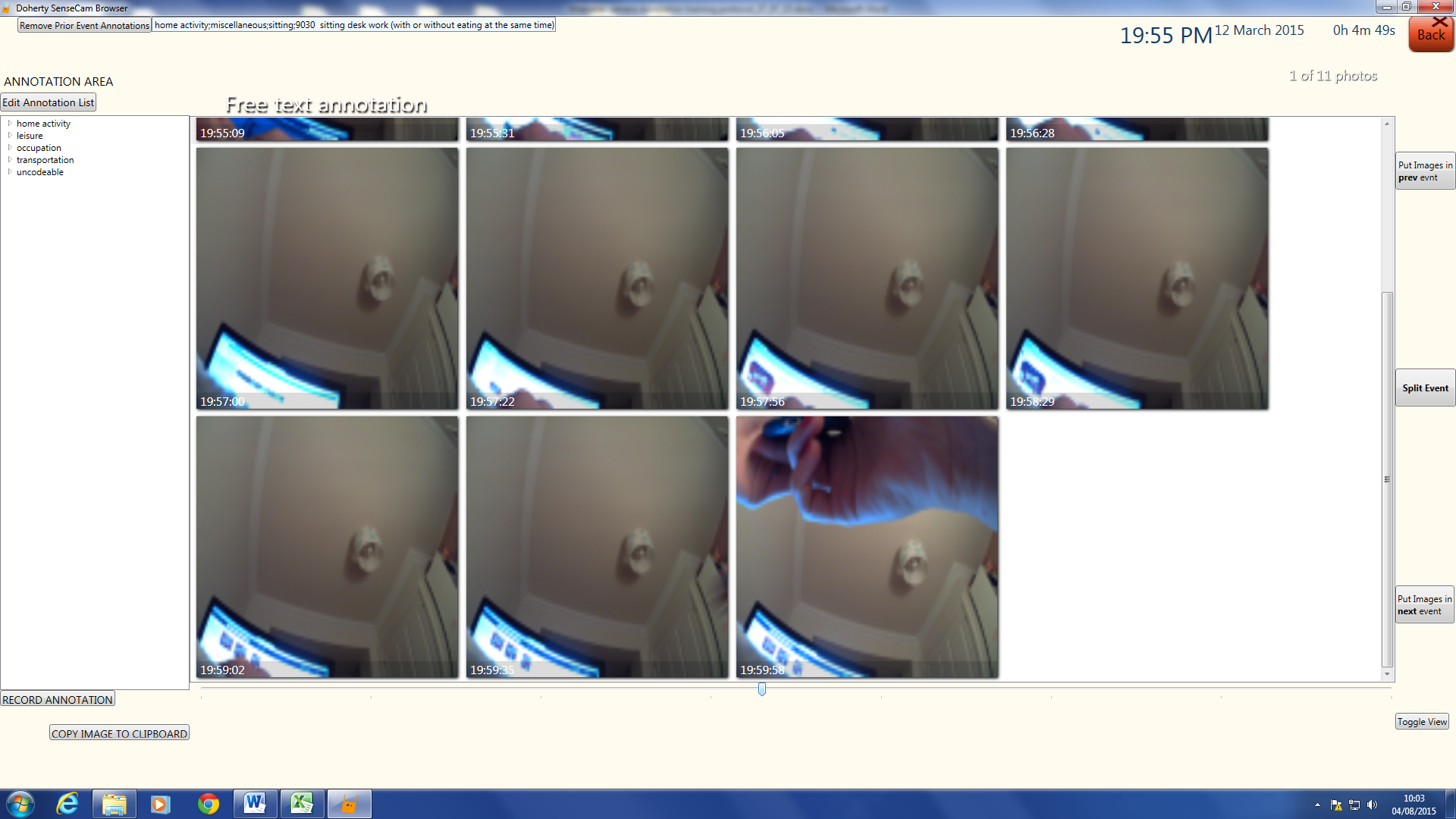

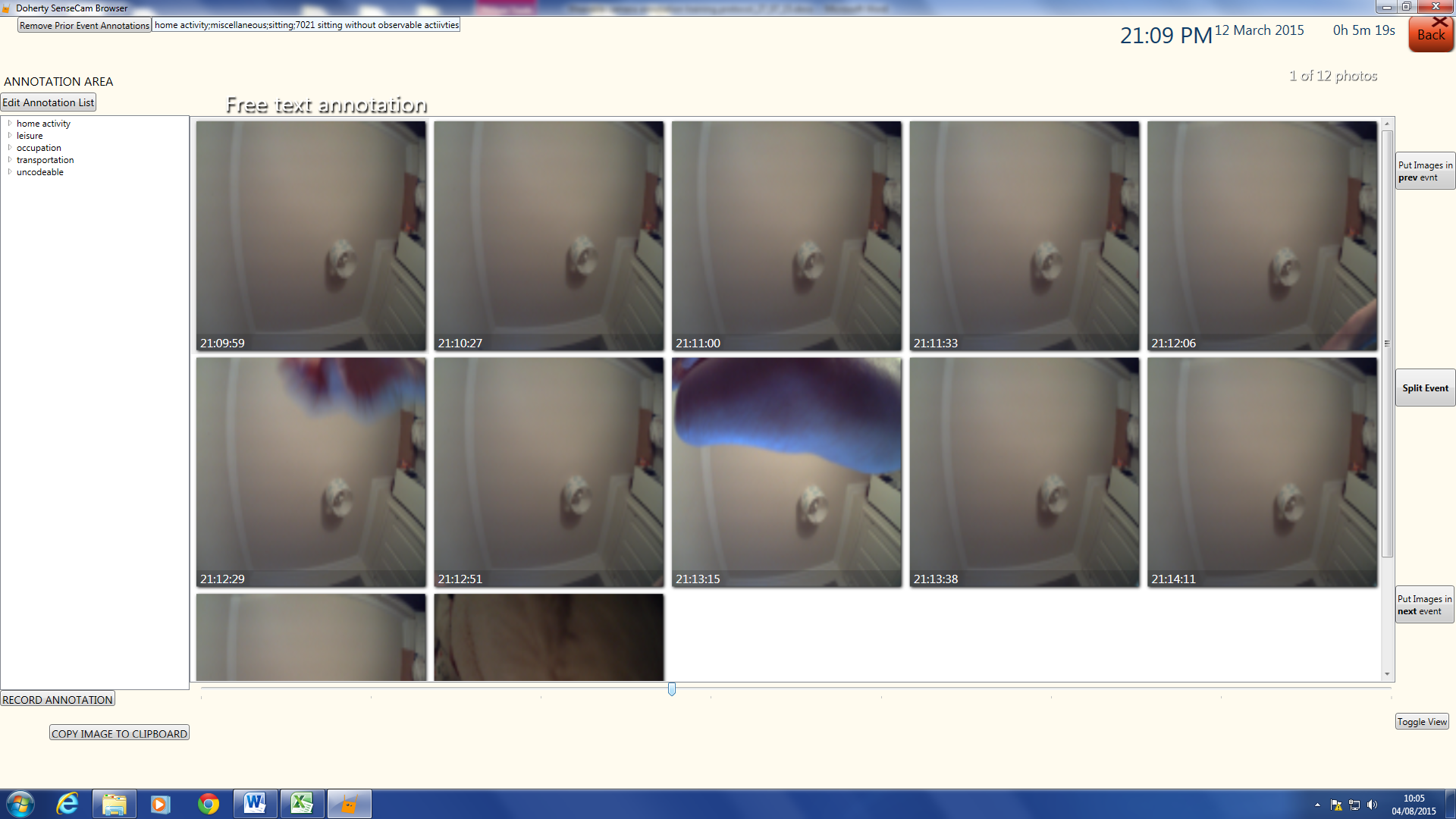

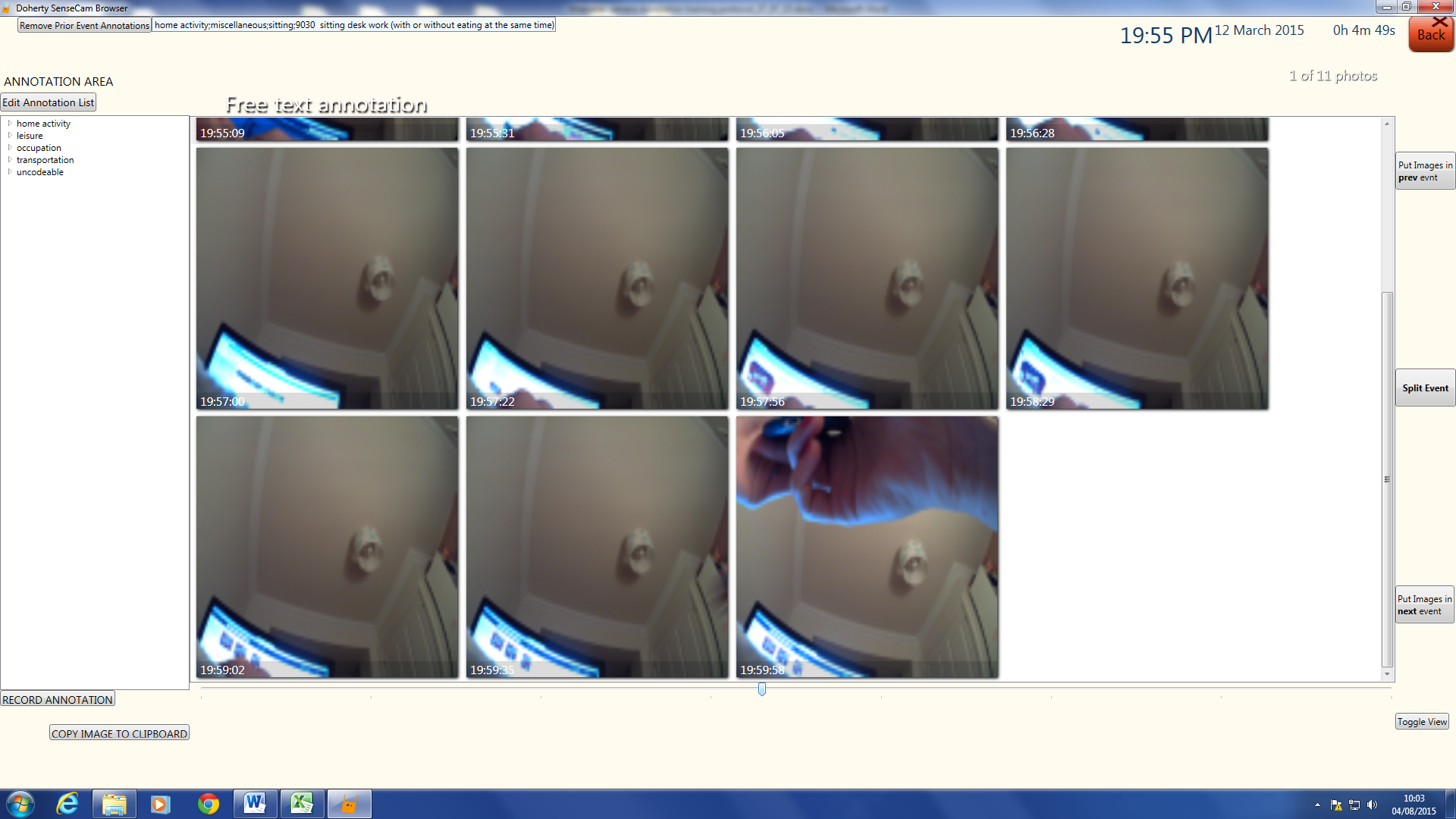

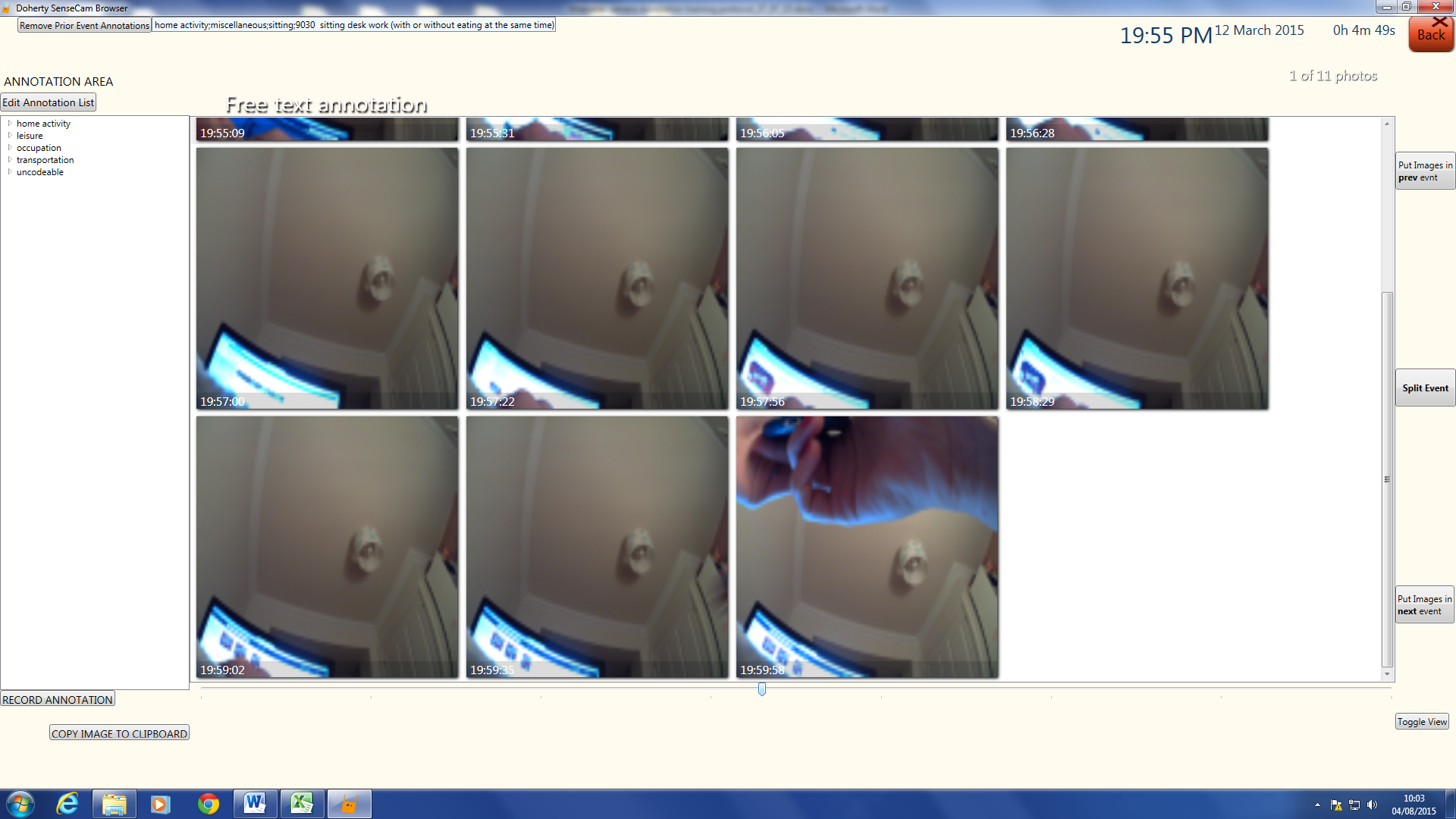

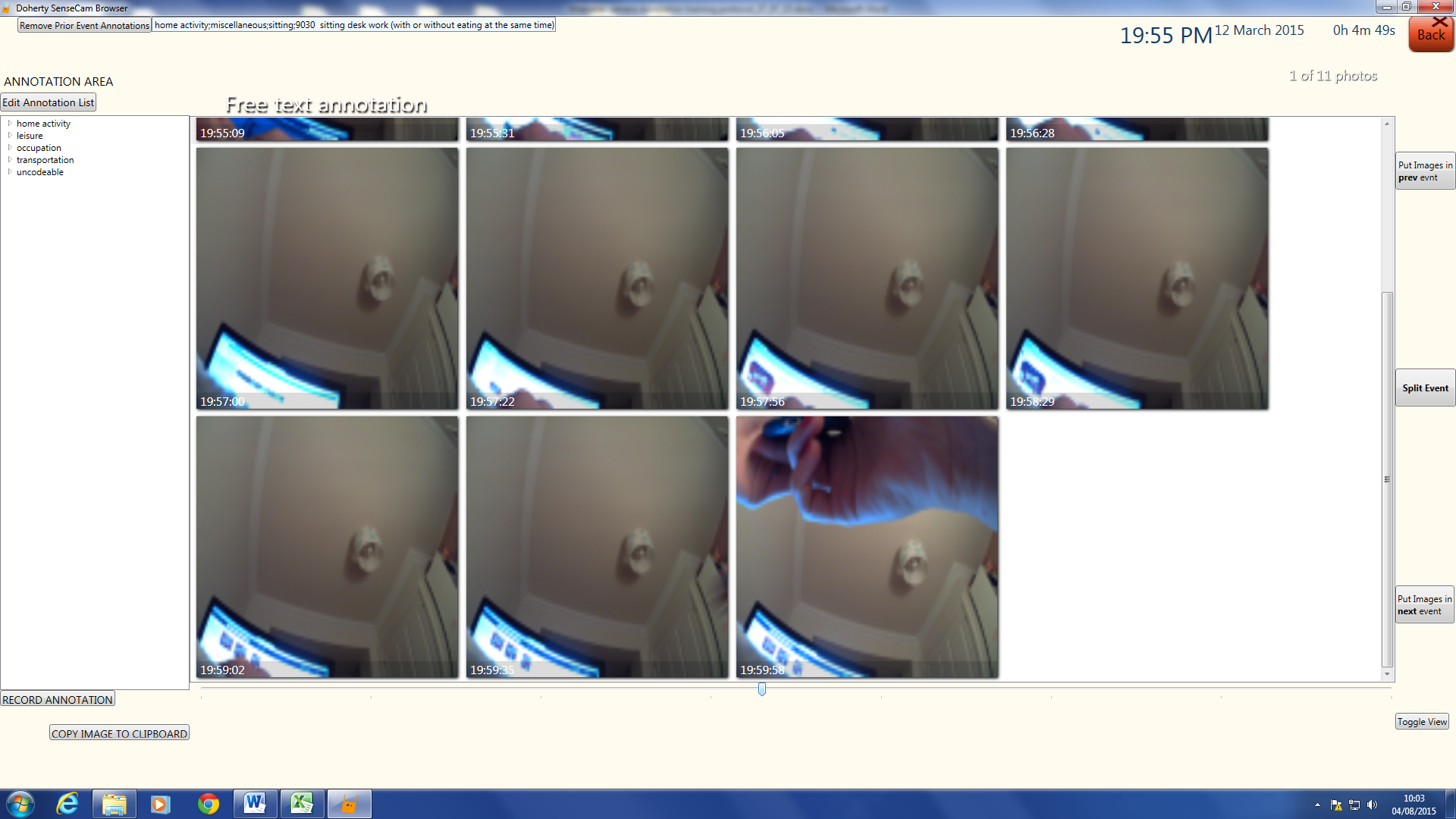

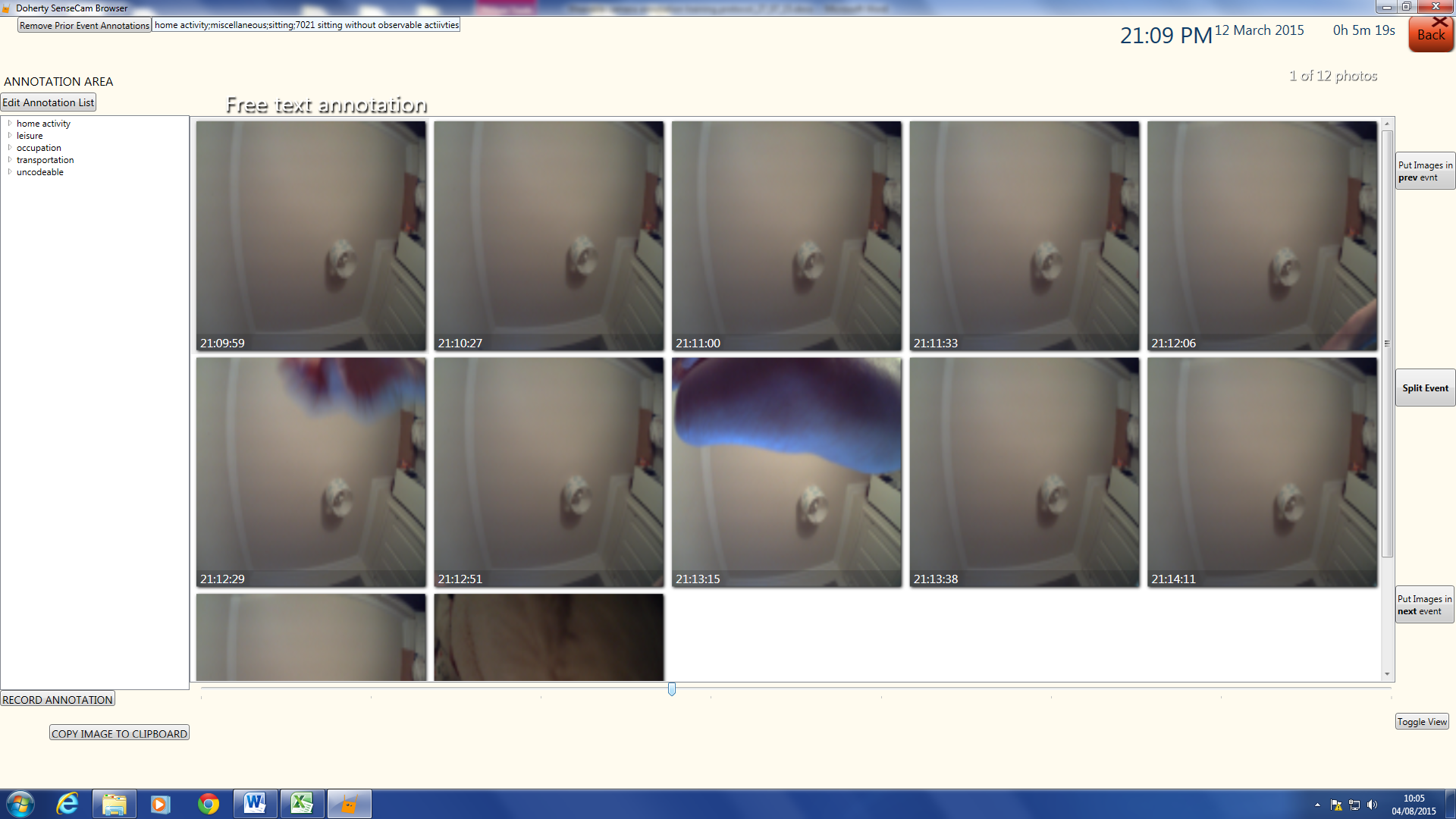


The first three images are “home activity; miscellaneous; sitting; 9060 sitting/lying reading or without observable/identifiable activities” and the following three “home activity; miscellaneous; sitting; 5080 sitting non-desk work (with or without eating at the same time)” as researchers cannot be certain whether the computer was on a desk. The five subsequent images are “home activity; miscellaneous; sitting; 9055 sitting/lying talking in person/using a mobile phone/smartphone/tablet or talking on the phone/computer (skype chatting),” as using the phone was the primary activity. The last five images are “home activity; miscellaneous; sitting; 9060 sitting/lying reading or without observable/identifiable activities”.

### **Case study 4. Teaching standing and discussion**

|  |
| --- |

In instances where the participant was standing teaching, the appropriate annotation is “occupation; public admin/education/health; education; 09071 teaching standing”. However, if the participant sat down and gave a tutorial to students the researcher should annotate the event as “occupation; interruption; 11585 sitting meeting/talking to colleagues with or without eating”, to give the most appropriate description of physical activity.


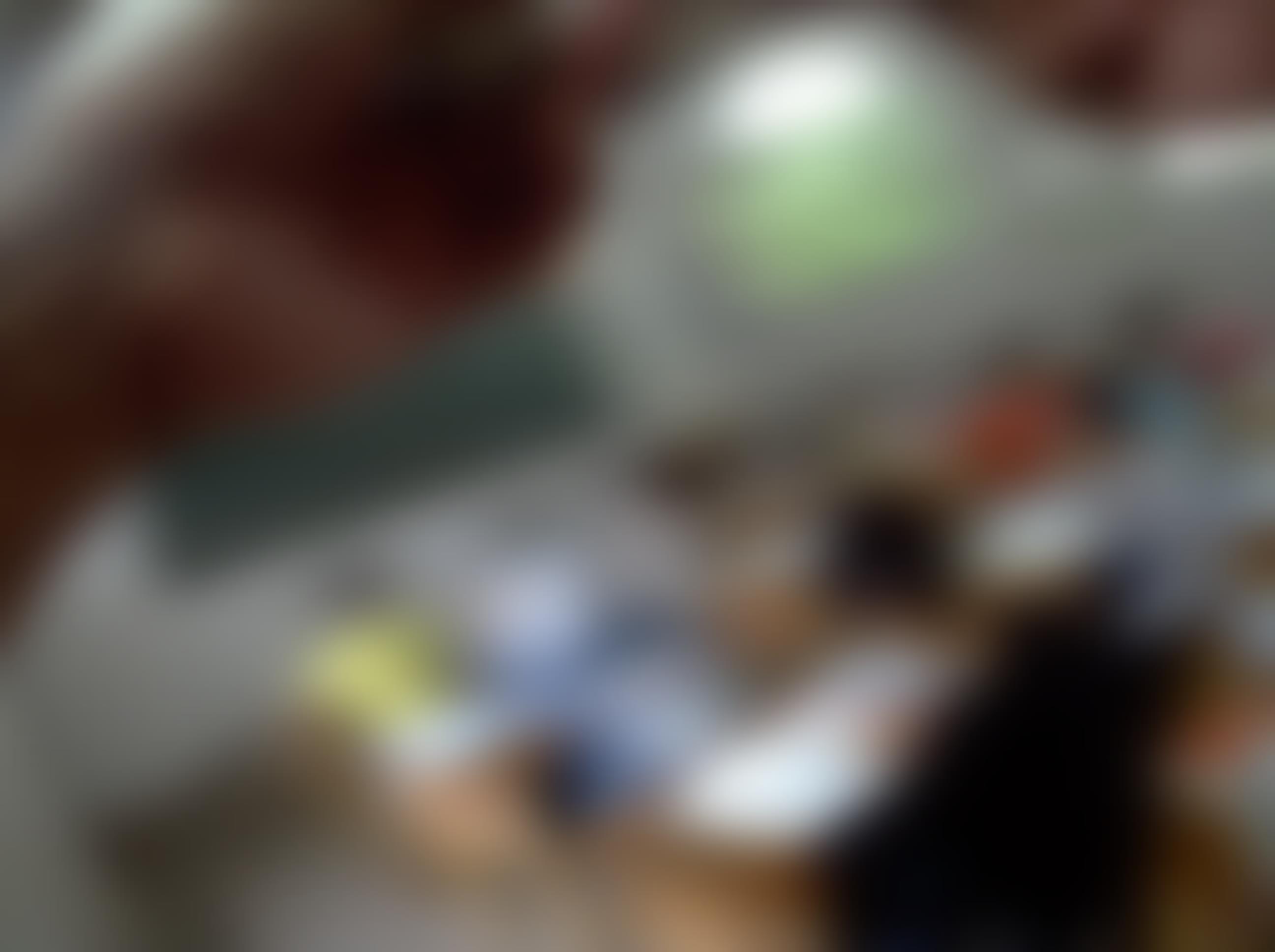

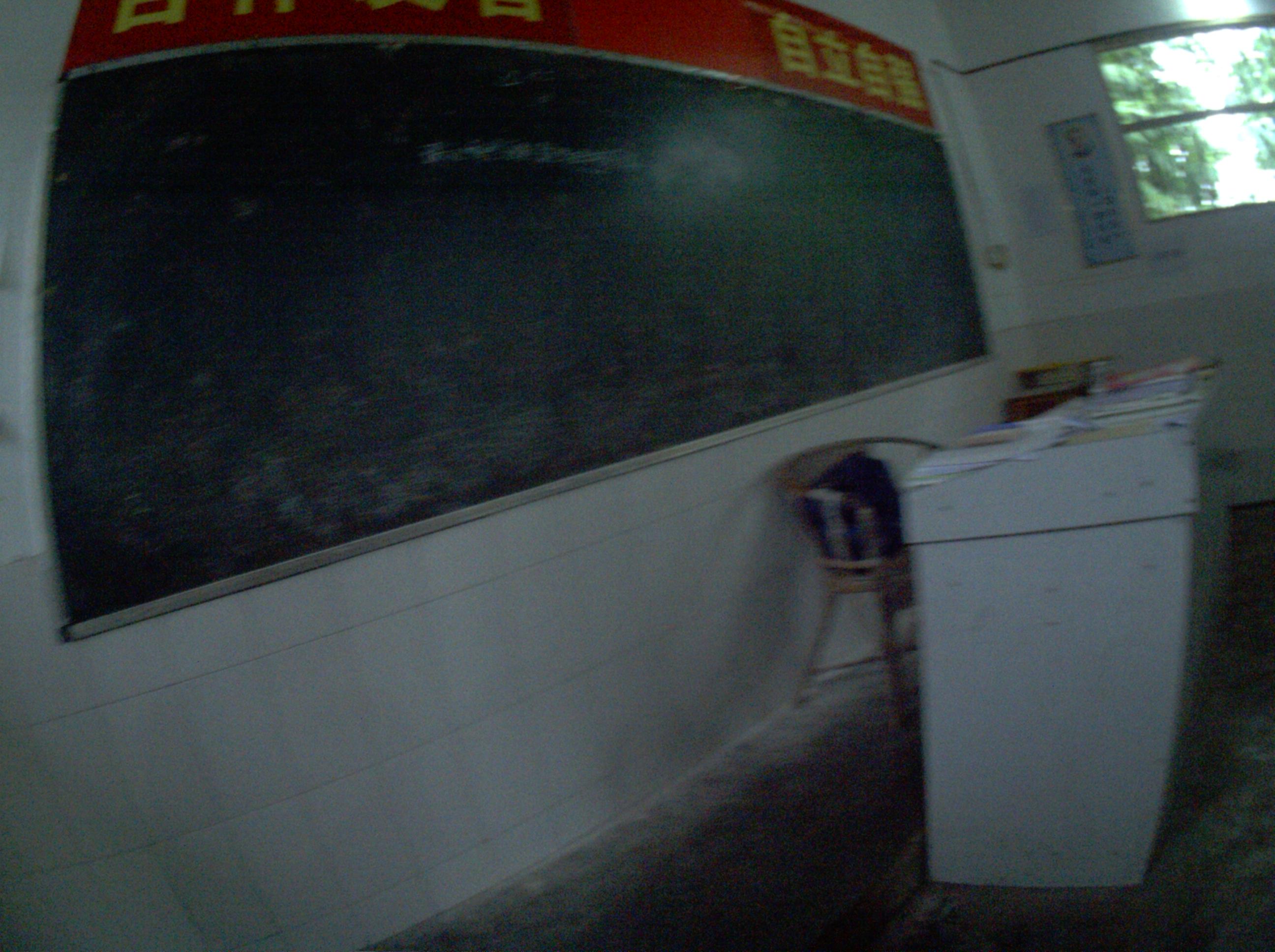

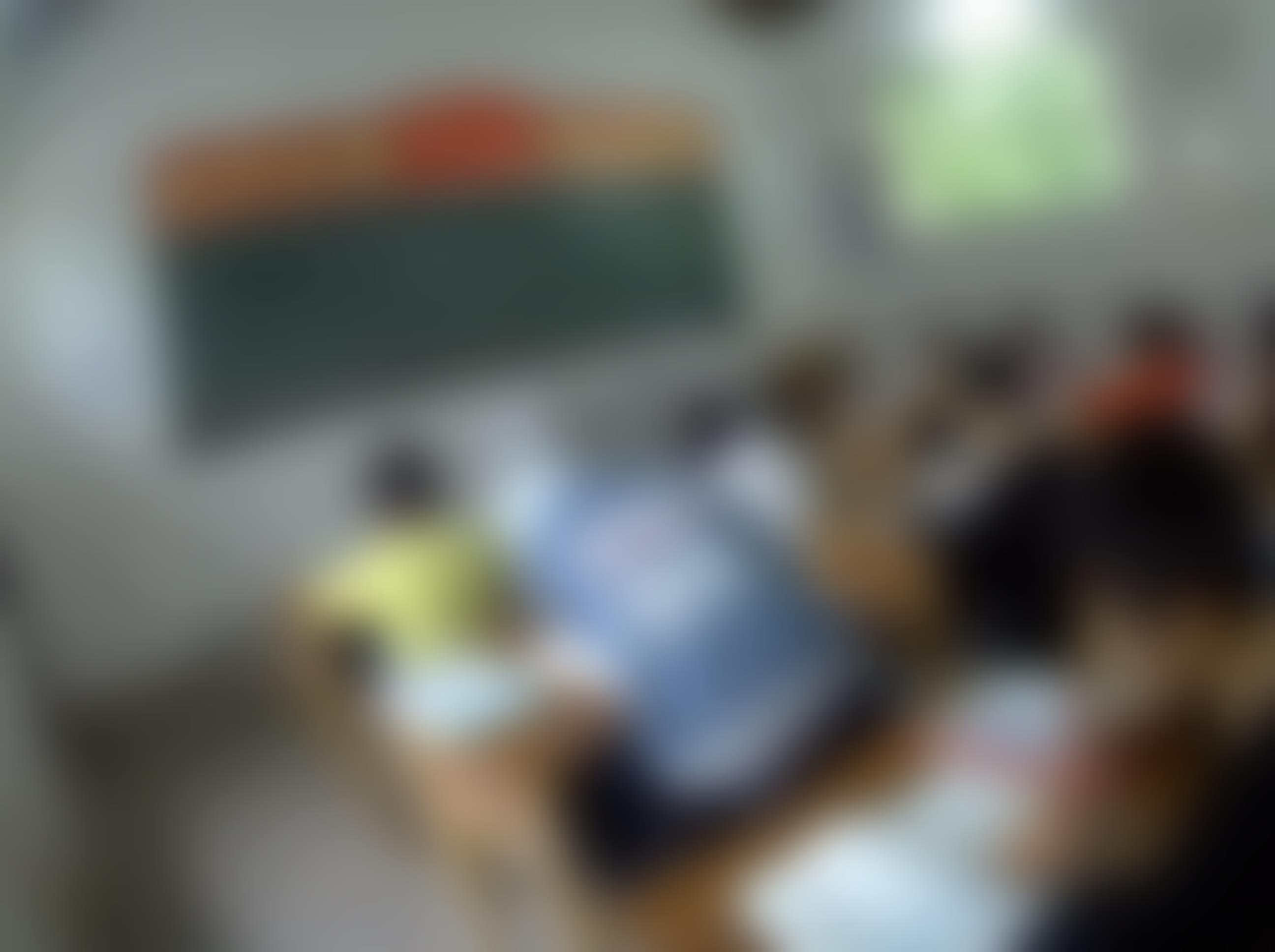

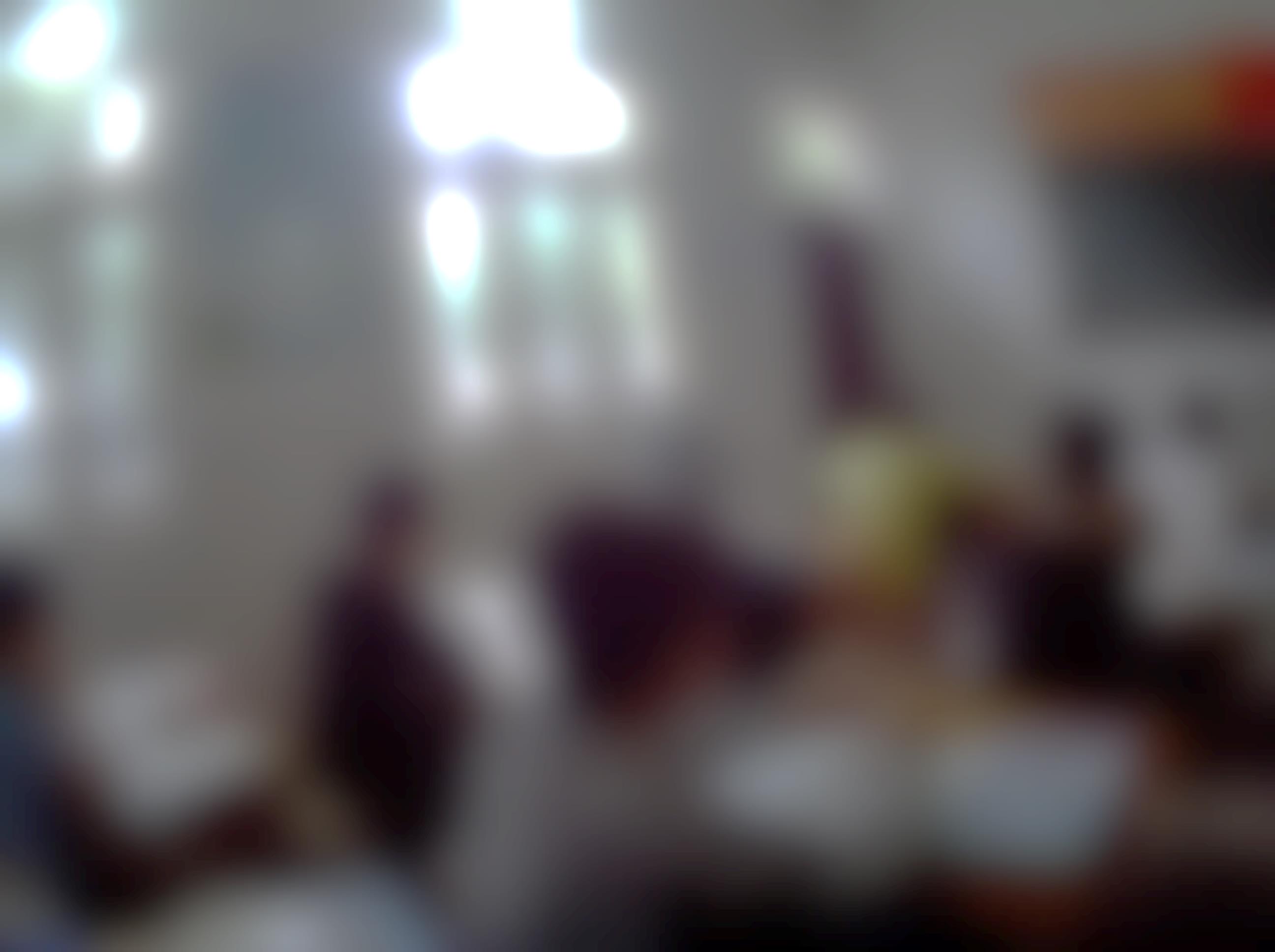

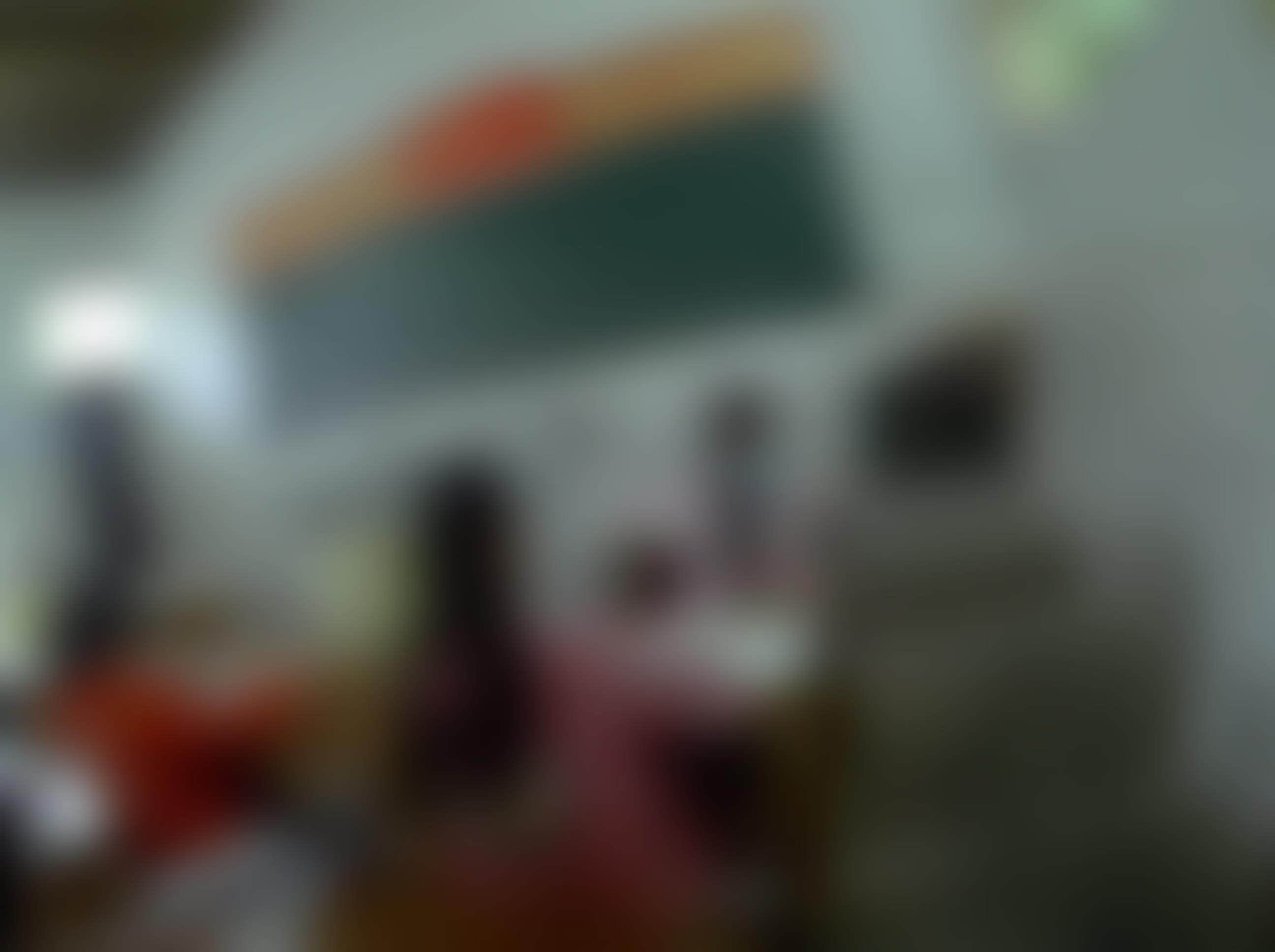

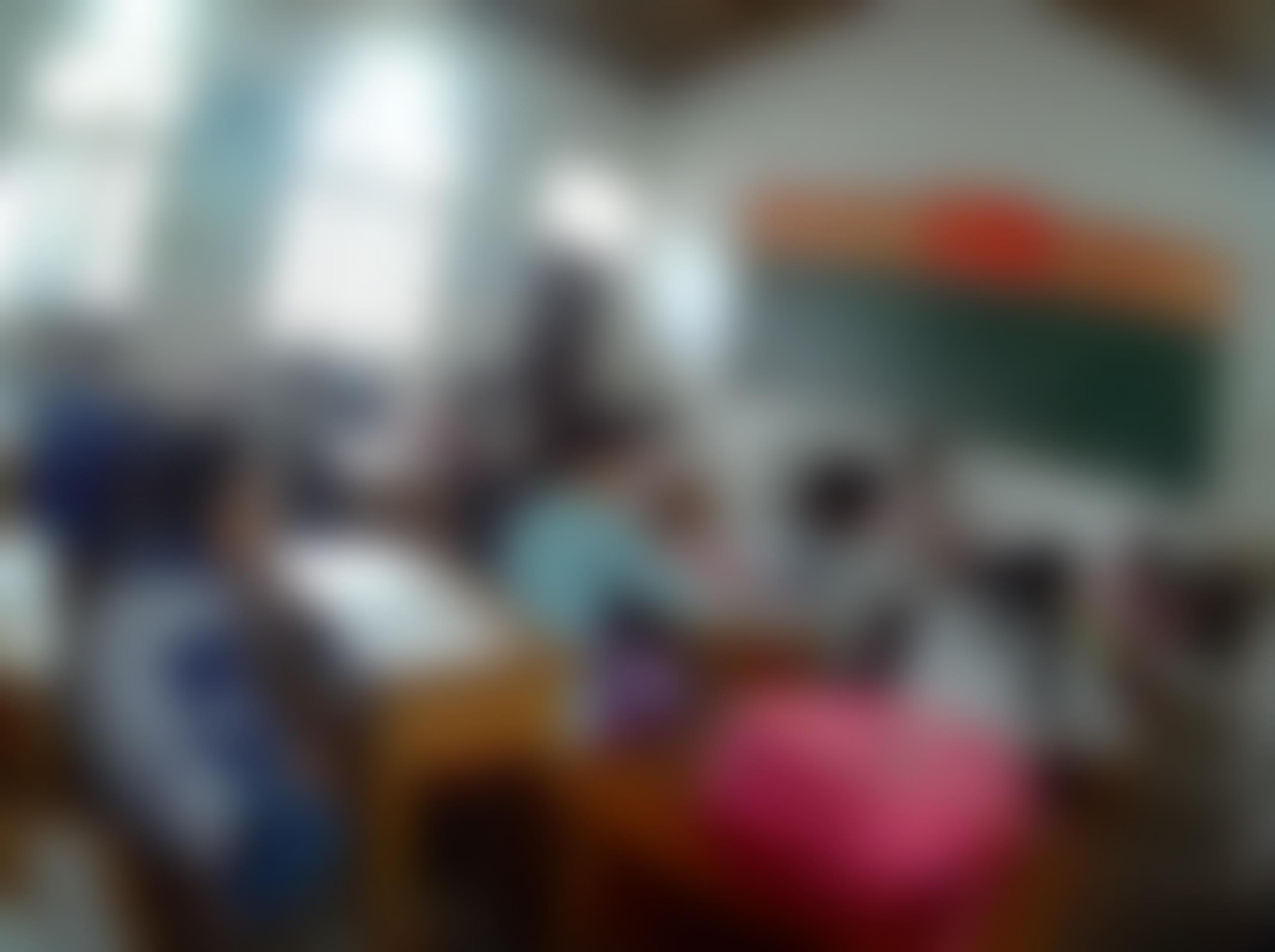

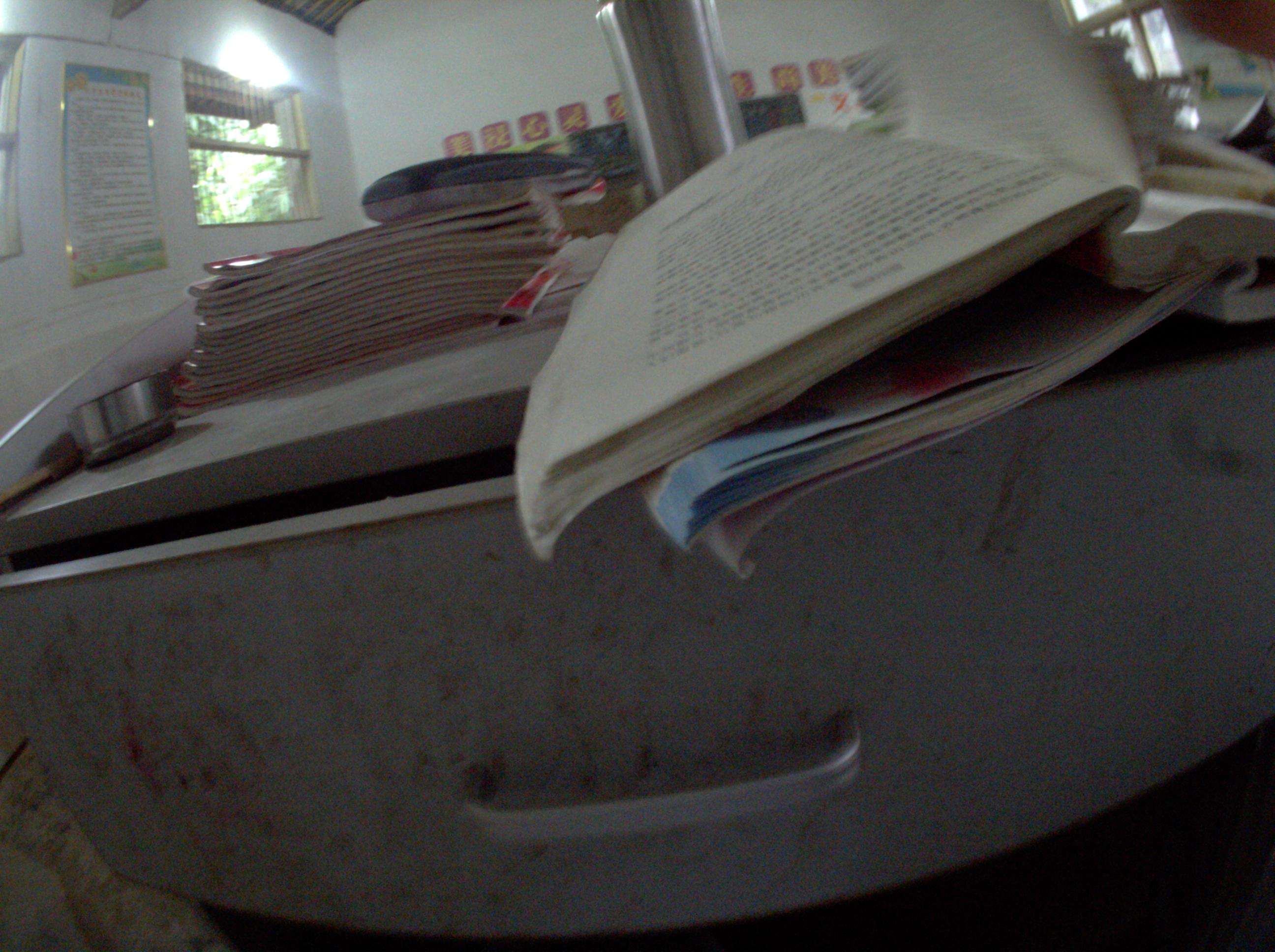

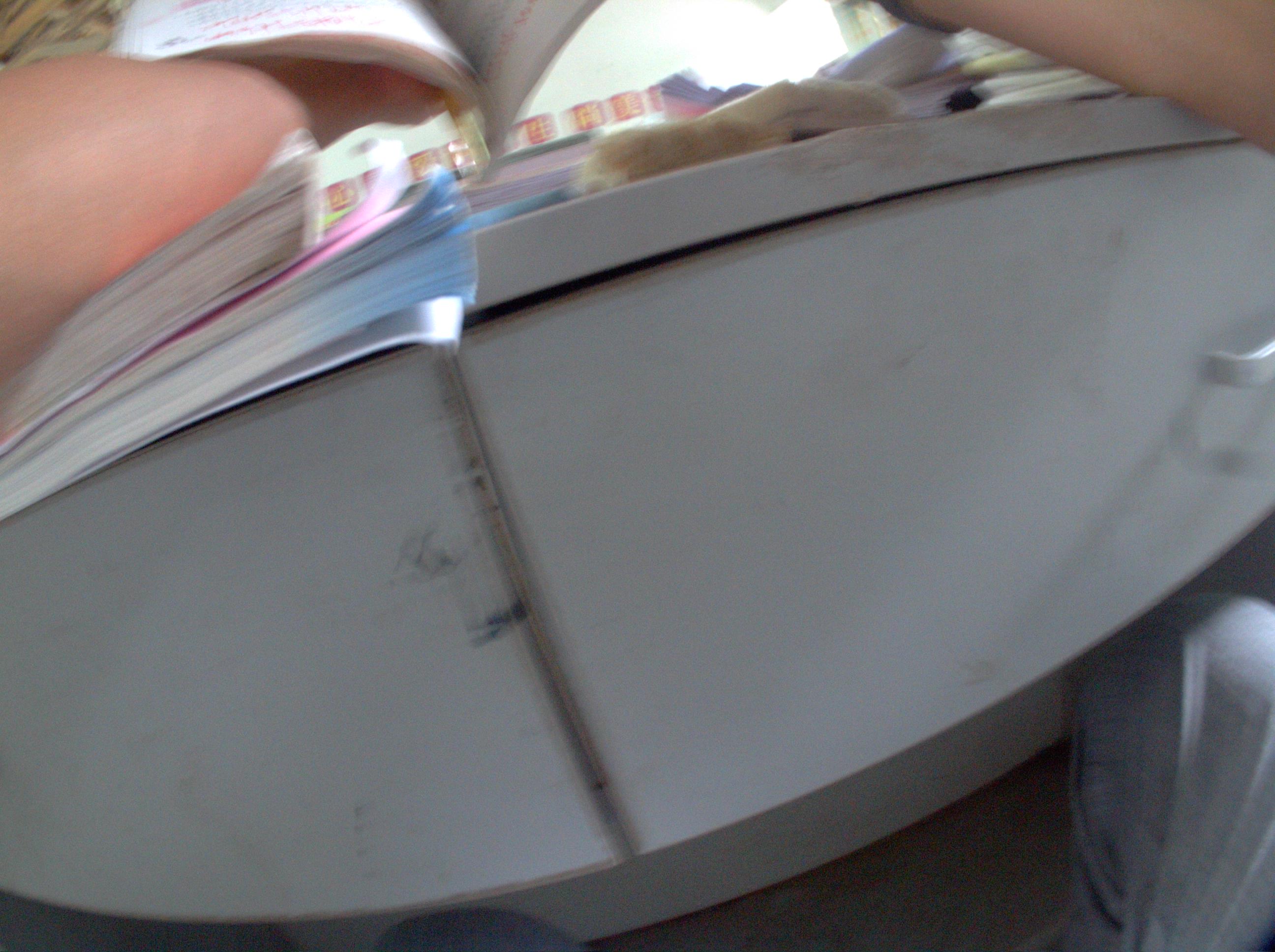

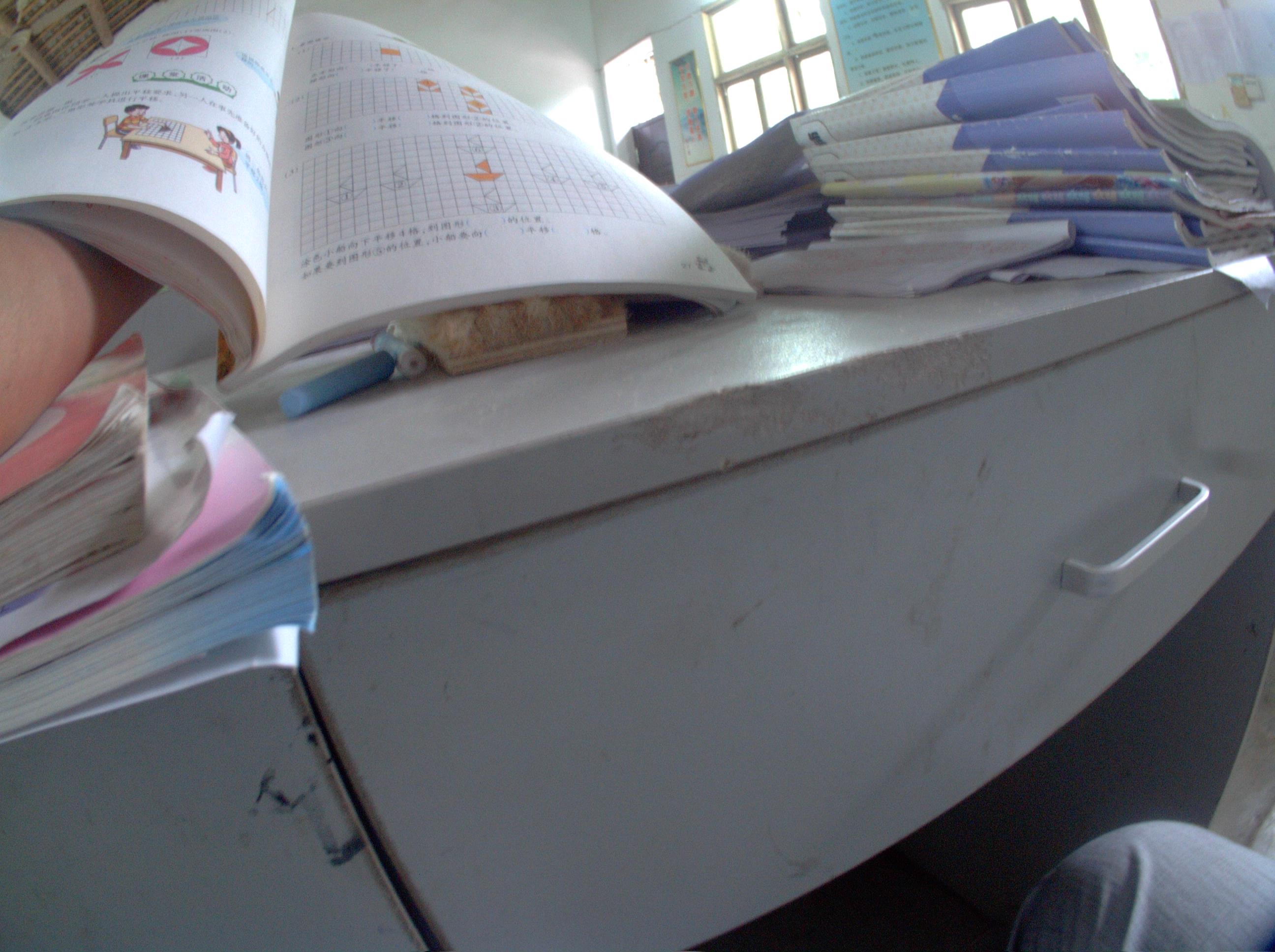

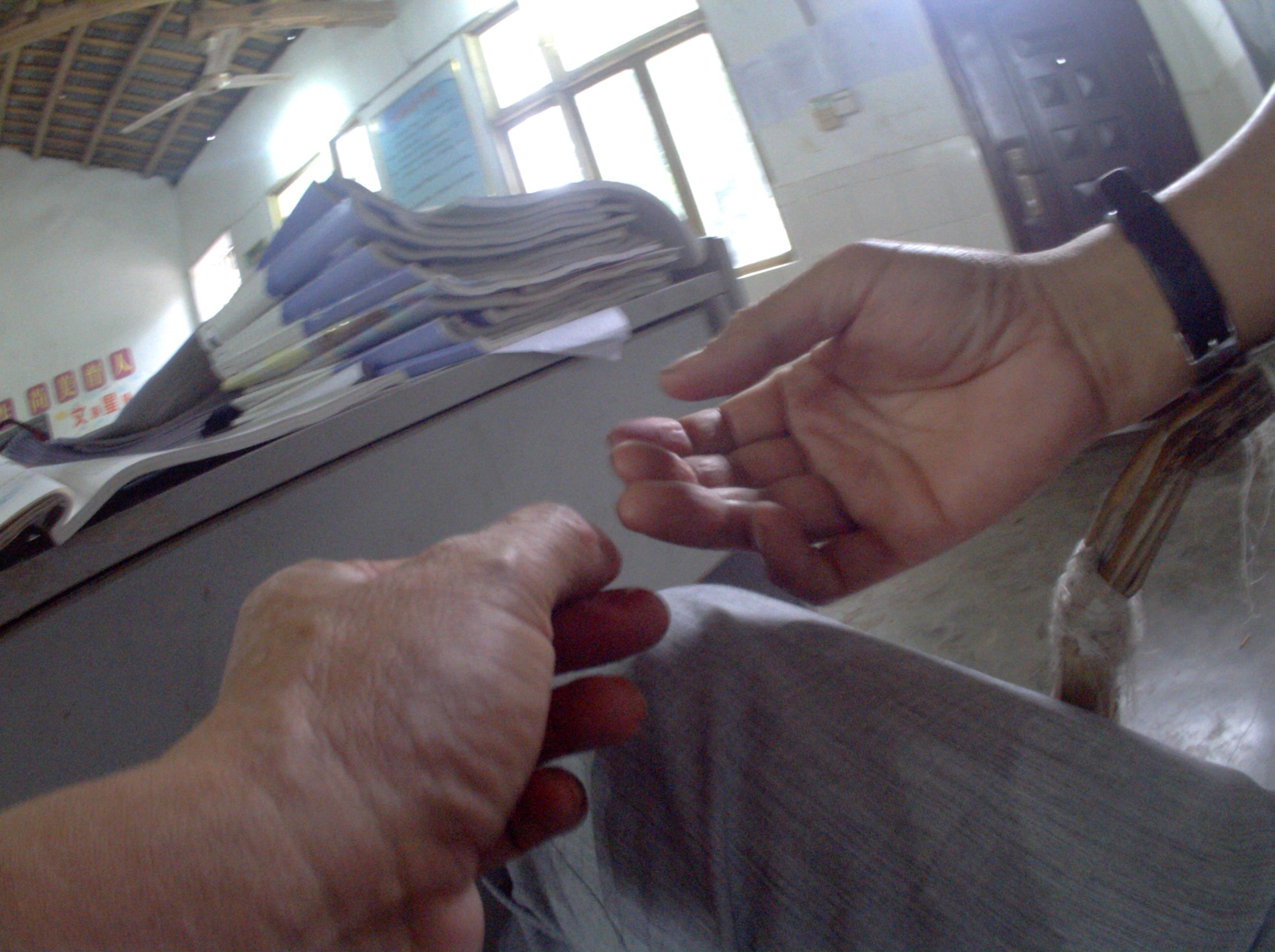

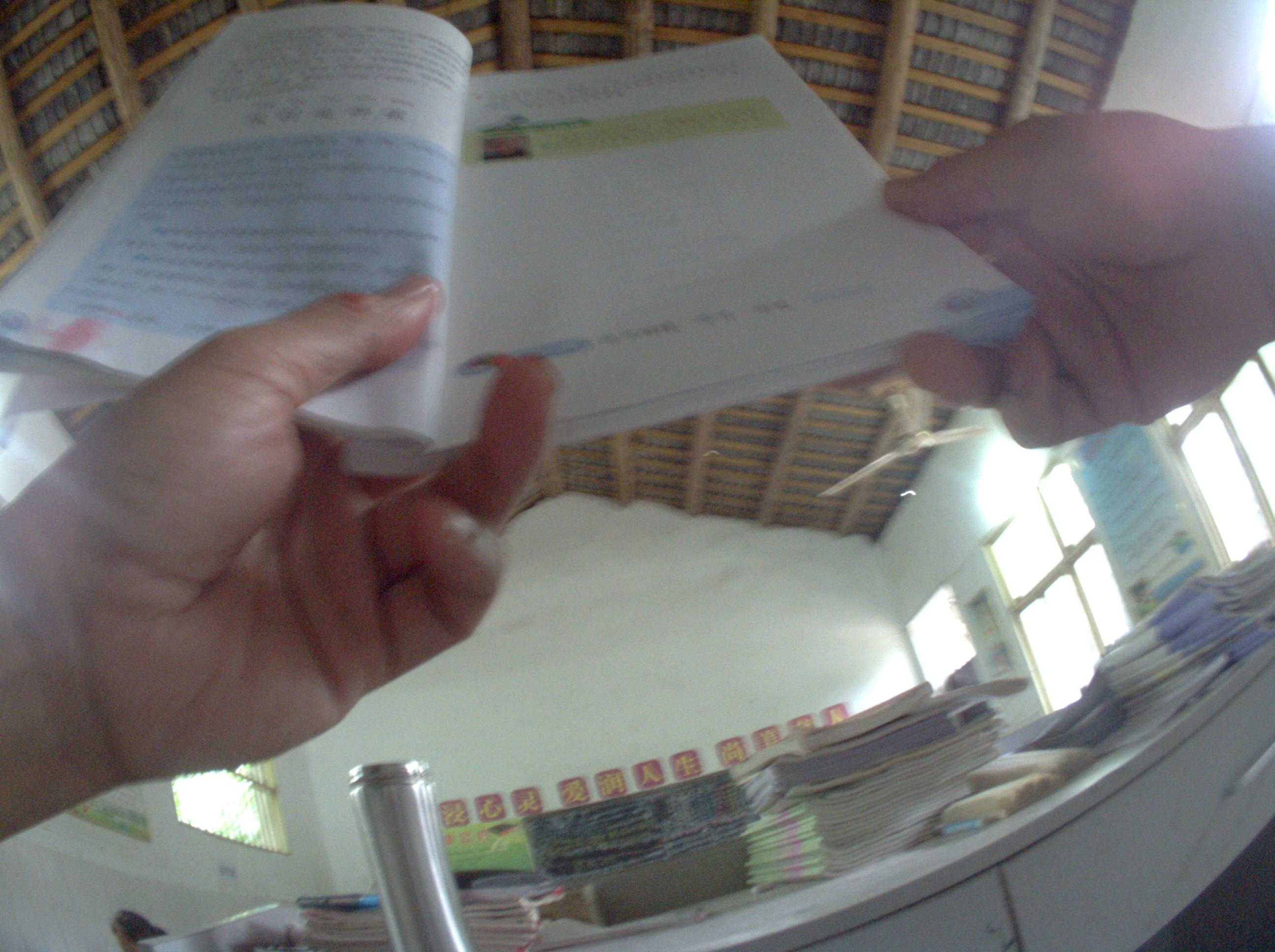

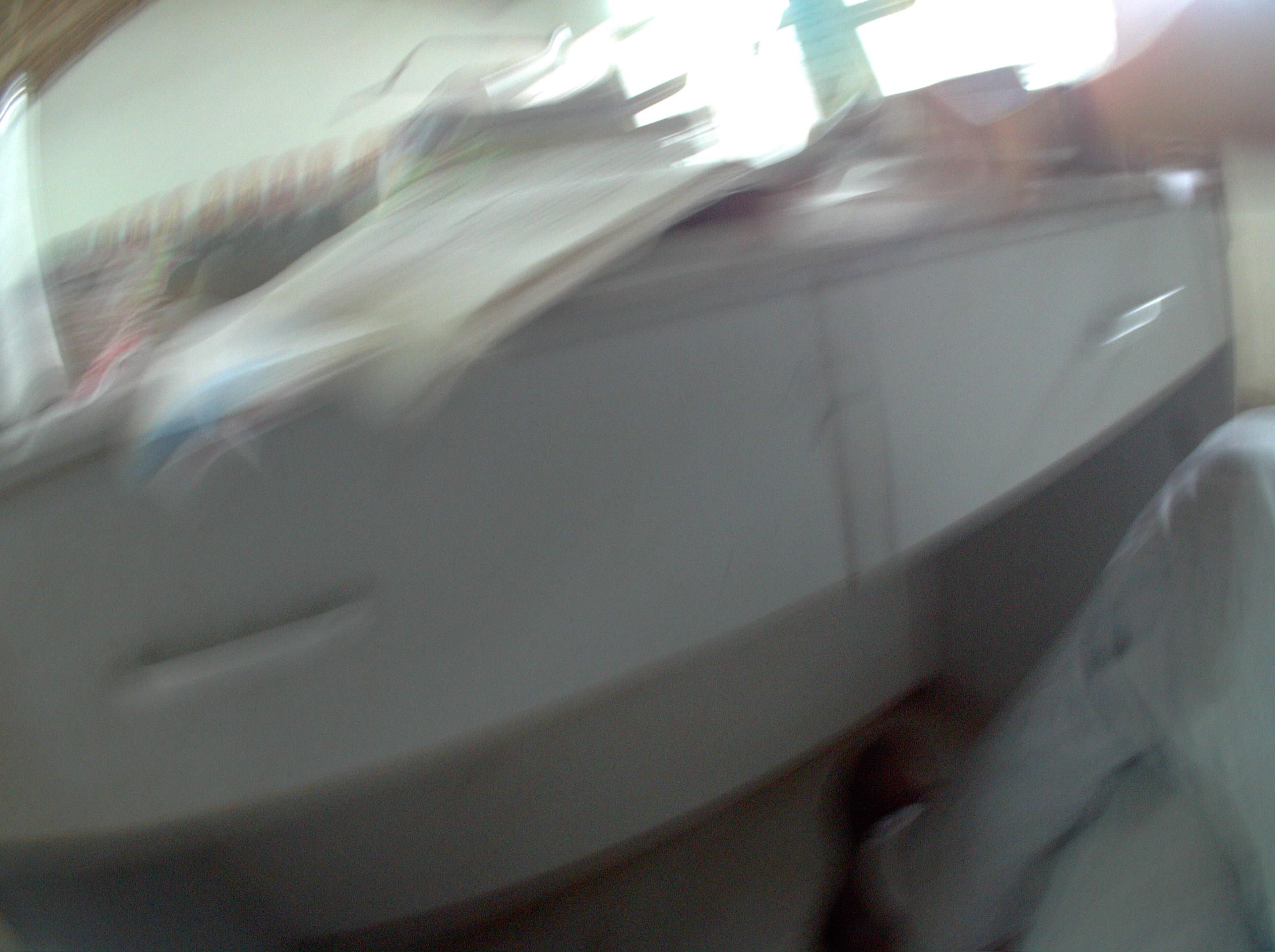


Here, the first 5 images would be annotated as “occupation; public admin/education/health; education; 09071 teaching standing”, and the remaining five images “occupation; interruption; 11585 sitting meeting/talking to colleagues with or without eating”.

### **Case Study 5. Office work and sitting activities without observable activities**


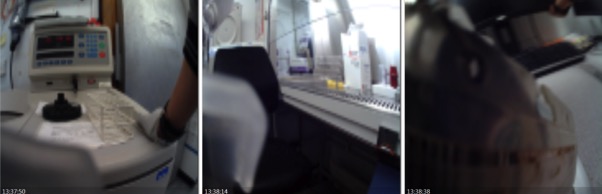


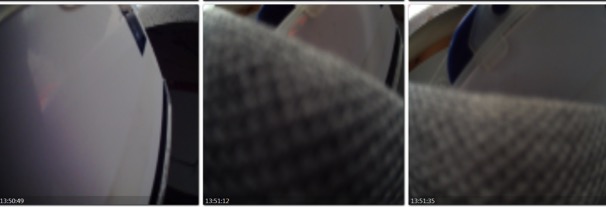


Here the participant was performing general laboratory work. For the first three images, the correct annotation is “occupation; office and administrative support; 11580 office work/computer work general”. However, as the camera was under the desk for the following three images, the appropriate annotation is “occupation; interruption; 9060 (generic) sitting without observable/identifiable activities”, as we cannot be certain as to what the participant was actually doing on the desk.

### **Case study 6. Standing without observable/identifiable activities and walking household without observable loads**


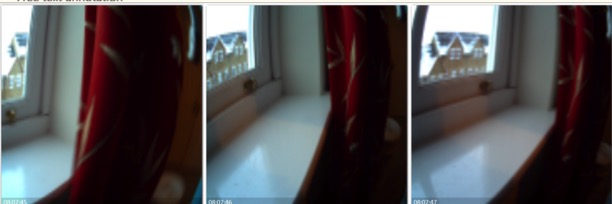


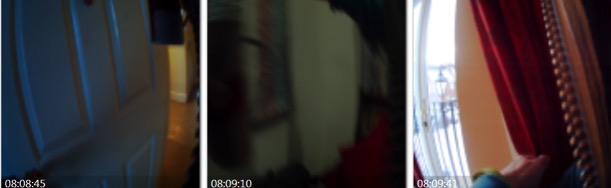


The first three images show that the participant was standing, yet the researcher isn’t able to identify what they are doing, so the event should be annotated as “home activity;miscellaneous;standing;9070 standing reading or without observable/identifiable activities”. For the remaining three, as the position and location of the participant kept changing, the correct annotation is “home activity; miscellaneous; walking; 17150 walking household without observable loads”.

### **Case study 7. Social settings/ eating and socialising**


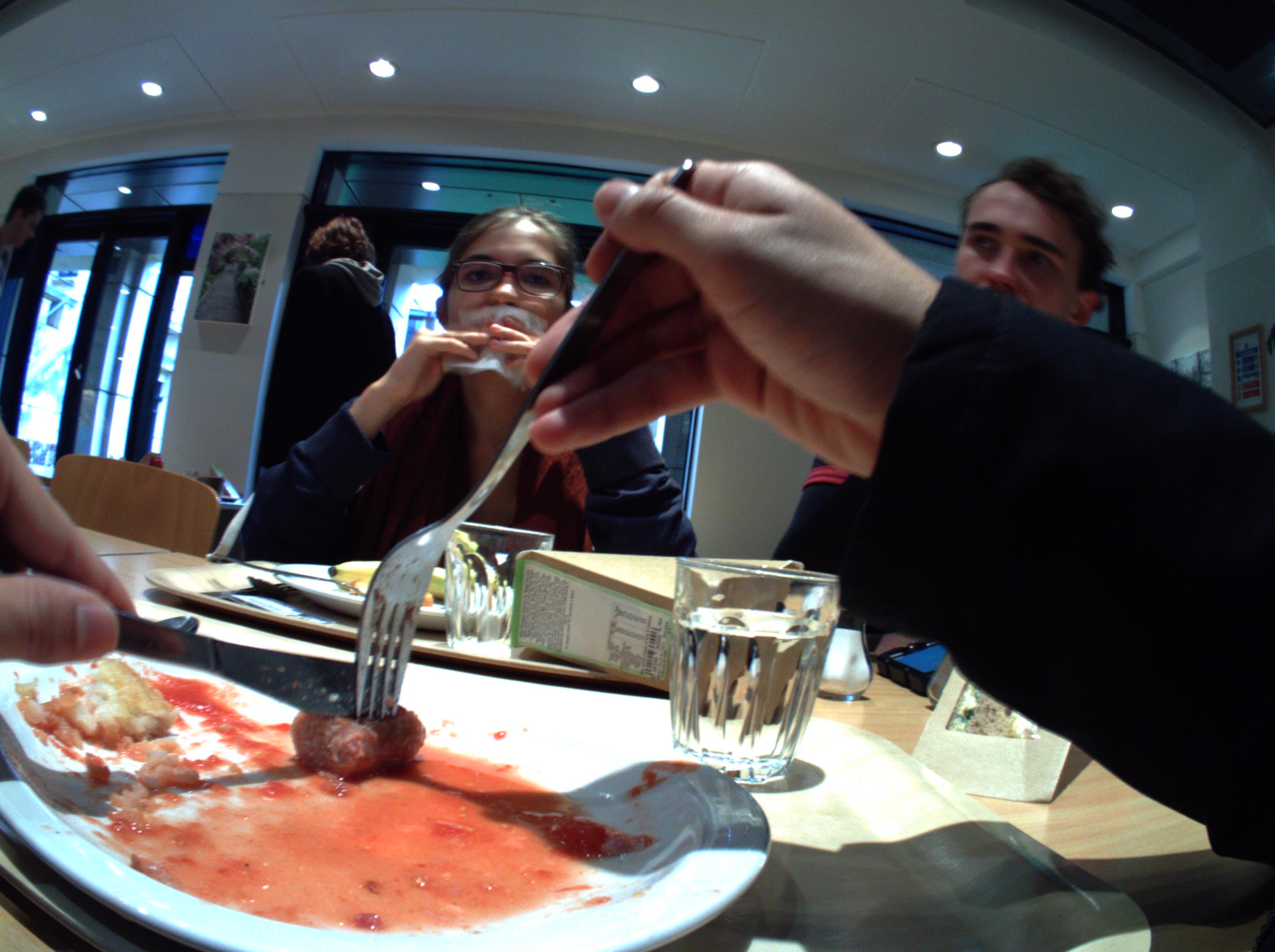

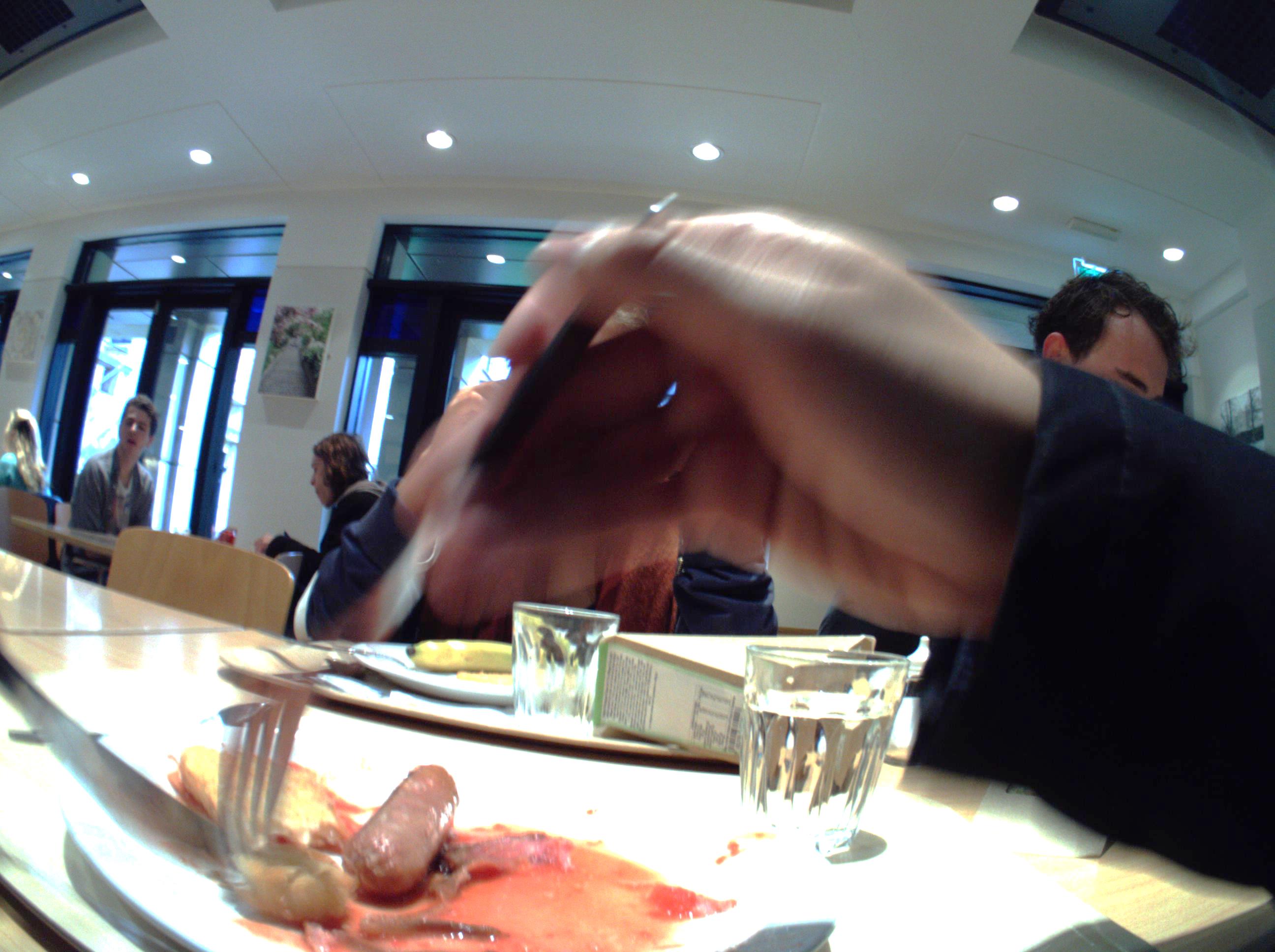

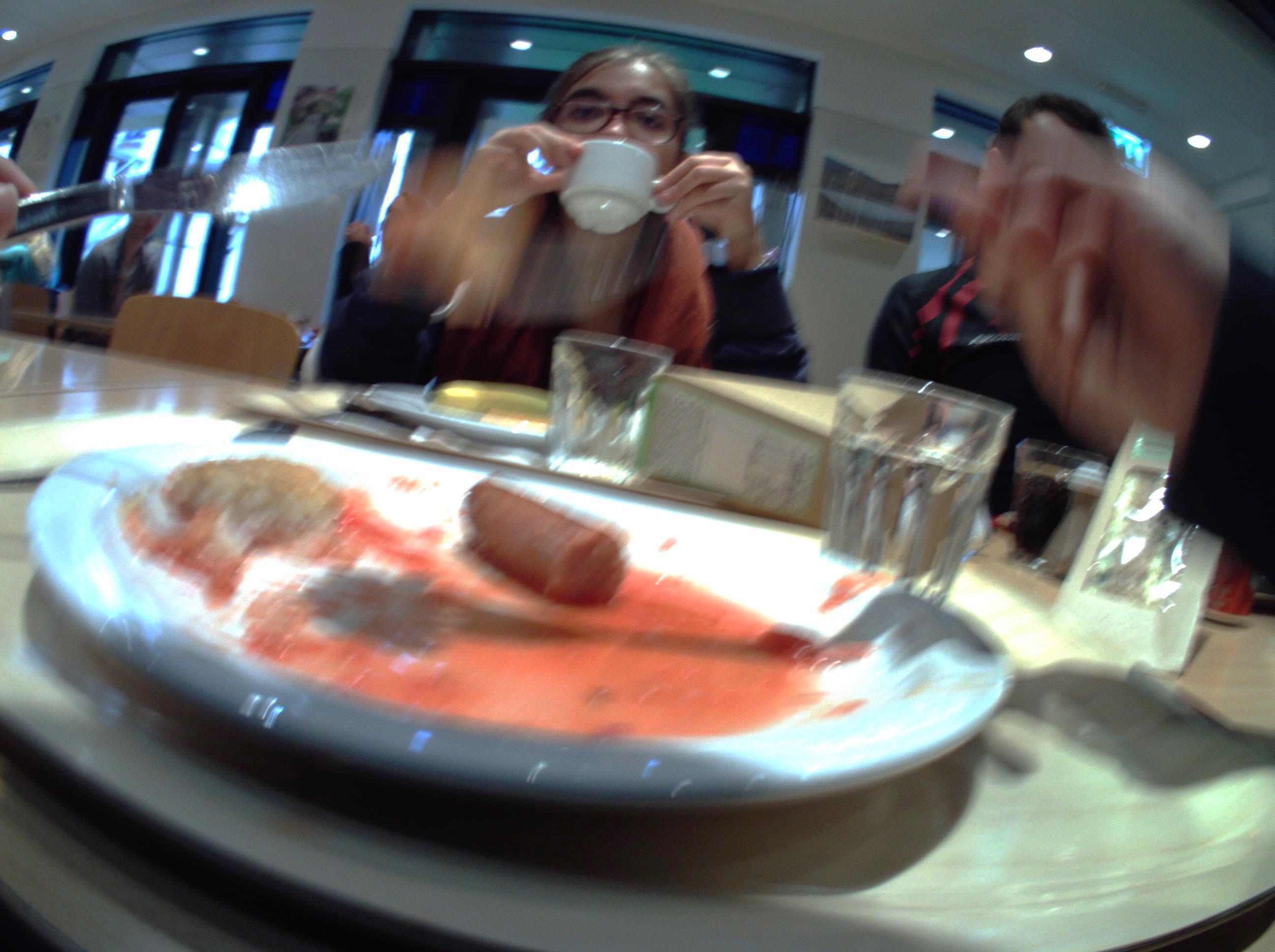

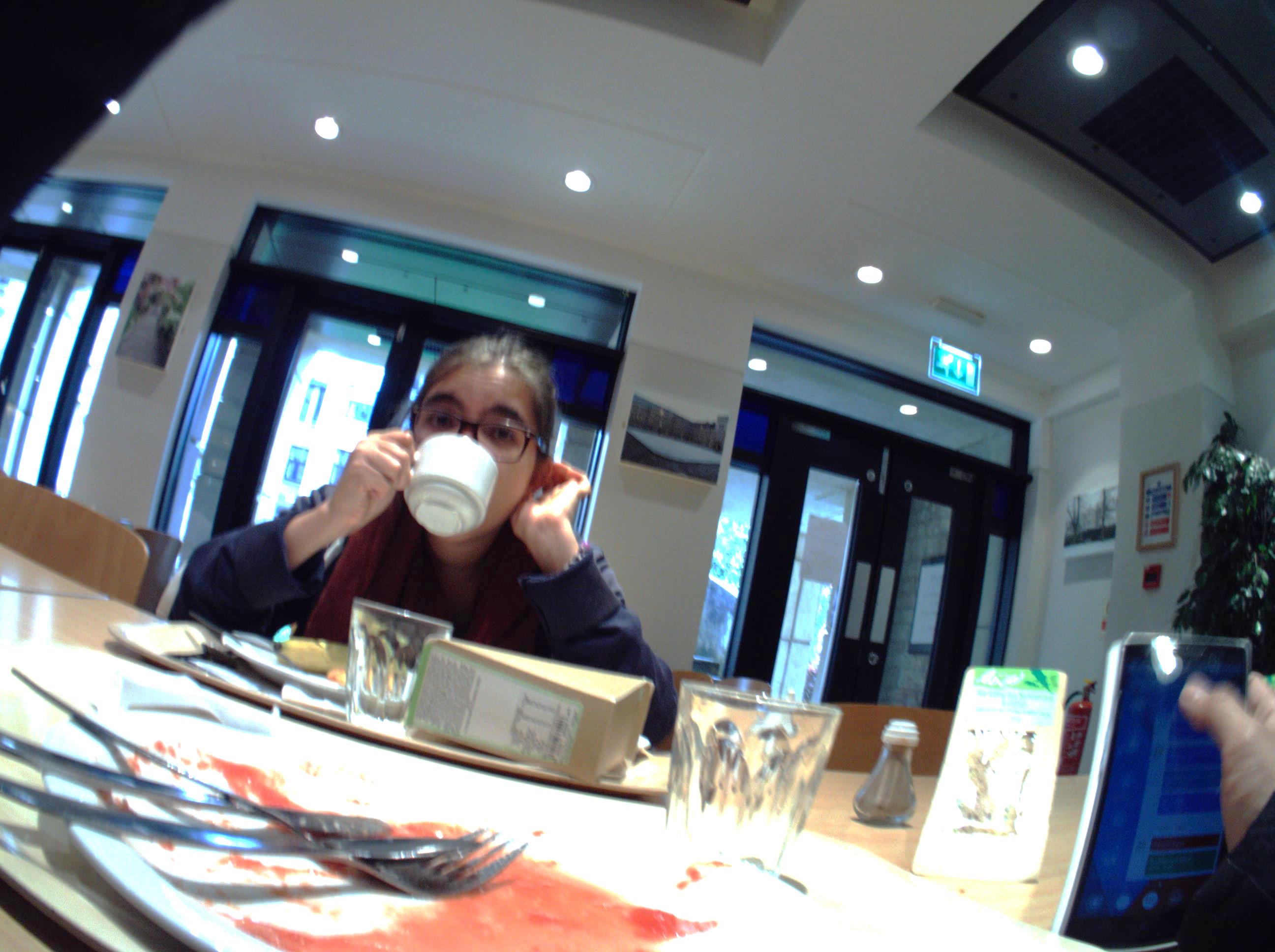

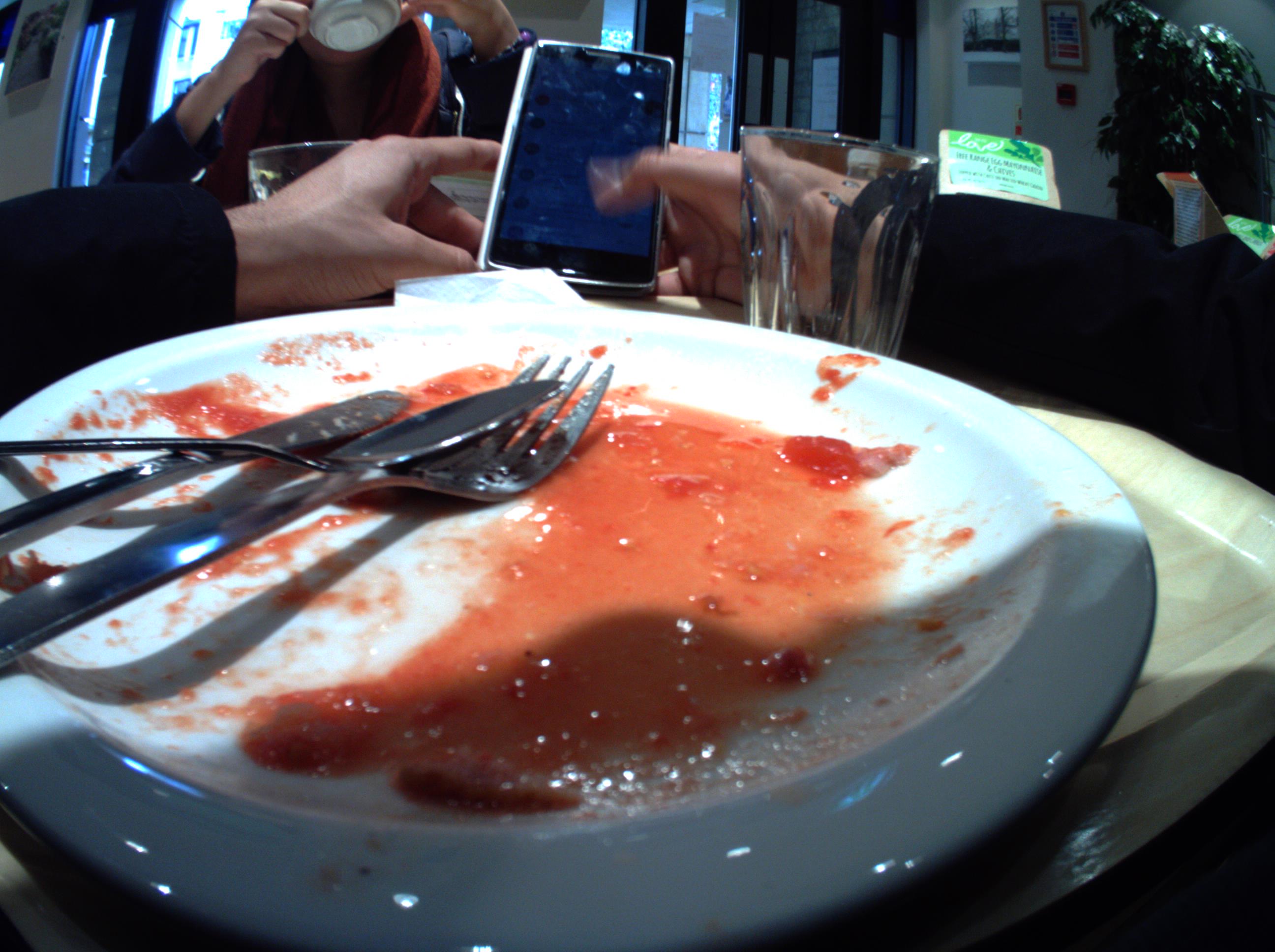

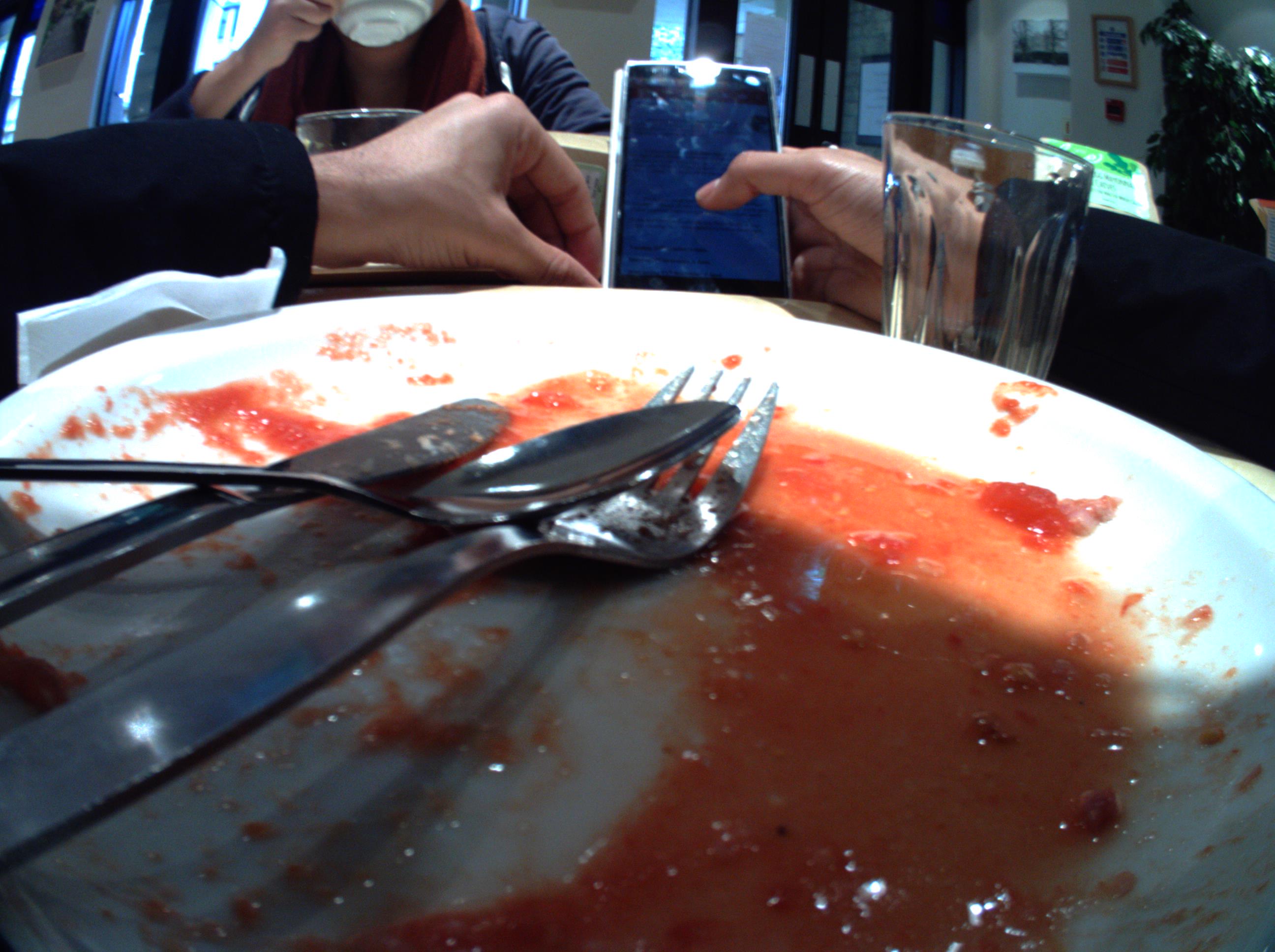


|  |  |  |
| --- | --- | --- |
|  |  |  |

The first three images show the participant sat at a table with food which they are clearly eating, therefore the correct annotation is “leisure; eating; 13030 eating sitting indoor/ outdoor”. However, in the following three consecutive images, the researcher is *sure* that the participant is no longer consuming food or drink- hence the appropriate annotation for the activity is “leisure; miscellaneous; sitting 9055 sitting talking to person/using the phone”.

This scenario often also occurs in a workplace, when the participant is on their lunch break with colleagues. Again when the participant may be eating the annotation used should be “occupation; interruption; sitting; 13030 eating sitting”, however when the participant is clearly not eating for at least 3 consecutive images, the correct annotation is “occupation; interruption; sitting; 11585 sitting meeting/ talking to colleagues with or without eating”.

# D. Quality checks

## Evaluating the validity of the annotation

In the context of annotating wearable camera images, annotation validity concerns the extent to which the researcher is accurately annotating what is actually occurring in the images. As we cannot be certain about what was actually occurring at the time the image was taken, we use an expert’s annotation as the proxy of the real activities. An expert’s judgement is still an opinion (albeit a good one) of a person’s position, context, or environment, we consider the level of agreement between a new researcher’s annotation and an expert’s annotation a measure of construct validity.

## Evaluating the reliability of a researcher

In the context of annotating wearable camera images, annotation reliability refers to:

1. intra-rater agreement: the extent to which a researcher is consistent within themselves (i.e. the extent to which they always annotate the same image in the same way); and
2. inter-rater agreement: the extent to which they agree with other researchers annotating the same image. Using these definitions you should see that validity is only a special case of inter-observer agreement (except that the “other researcher” is an expert).

Both forms of reliability should be evaluated. To measure intra-rater agreement, the researcher should annotate the same group of images twice, one week apart. To measure inter-rater agreement, the group of researchers being used should periodically be asked to annotate the same images (preferable a random sample). If these have been annotated by an expert, this can be considered a test of validity. Otherwise, this is a measure of inter-rater agreement.

## Guidelines for Inter-rater agreement test

Inter-Rater Agreement tests (IRR) should be performed for validity and reliability tests using the programming language R. Training requires that a researcher to annotate up to nine sample sets of images pre-annotated by an expert with the Cohen’s kappa score in the inter-rater agreement test of 0.**80** or higher. Attempters who do not pass the training test must be re-trained and re-tested.

1. Ainsworth, B. E. *et al*. (2011). Compendium of Physical Activities: a second update of codes and MET values. *Medicine and Science in Sports and Exercise, 43* (8), 1575-1581. [↑](#endnote-ref-1)
2. United State Department of Labour. (2015). Standard Industrial Classification (SID) division structure. [WWW]. Retrieved from <https://www.osha.gov/pls/imis/sic_manual.html> [↑](#endnote-ref-2)
3. Office of National Statistics. (2011). Labour force survey background and methodology 2011. [WWW]. Retrieved from <http://www.ons.gov.uk/ons/guide-method/method-quality/specific/labour-market/labour-market-statistics/volume-1---2011.pdf> [↑](#endnote-ref-3)
4. Askari, S. F., Erchull, M. L., Staeball, S. E., & Axelson, S. J. (2010). Men want equality, but women don’t expect it: Young adults’ expectations for participation in household and child care chores. *Psychology of Women Quarterly, 34* (2)*,* 243-252. Retrieved from <http://onlinelibrary.wiley.com/doi/10.1111/j.1471-6402.2010.01565.x/epdf> [↑](#endnote-ref-4)
5. Kroska, A. (2003). Investigating gender differences in the meaning of household chores and child care. *Journal of marriage and family, 65* (2), 456-473. Retrieved from <http://onlinelibrary.wiley.com/doi/10.1111/j.1741-3737.2003.00456.x/epdf> [↑](#endnote-ref-5)
6. Bureau of Labour Statistics. (2015). American time use survey—2014 result. [WWW]. Retrieved from <http://www.bls.gov/news.release/pdf/atus.pdf>

   ^vii^ Doherty, A. R., Moulin, C. J. A. & Smeaton, A. F. Automatically assisting human memory: A SenseCam browser. *Memory* **19,** 785–795 (2011). [↑](#endnote-ref-6)
