# Supplementary material for: Author Correction: Statistical machine learning of sleep and physical activity phenotypes from sensor data in 96,220 UK Biobank participants
Source: Sci Rep. 2022 Apr 11;12:6024. doi: 10.1038/s41598-022-10148-5 (PMC9001648; doi:10.1038/s41598-022-10148-5)
Supplement: Supplementary file 1 — Supplementary Information. [file 41598_2022_10148_MOESM1_ESM.docx]

**Statistical machine learning of sleep and physical activity phenotypes from sensor data in 96,220 UK Biobank participants.**

**Matthew Willetts, Sven Hollowell, Louis Aslett, Chris Holmes, Aiden Doherty**

# Supplementary Tables / Figures

**Table. S1.** Differences between confusion matrices: machine learned behaviours classified from wrist-worn accelerometer data when using Hidden Markov Models versus not: the CAPTURE-24 study 2014-2015 (n = 132).

1. Random Forest Only *(unit = minutes of behaviour)*

| Ground truth→  Prediction↓ | **sleep** | **sit/stand** | **vehicle** | **walking** | **mixed-activity** | **bicycling** |
| --- | --- | --- | --- | --- | --- | --- |
| **sleep** | **65,906** | 3,087 | 22 | 17 | 28 | 13 |
| **sit/stand** | 6,389 | **37,917** | 833 | 1,162 | 1,616 | 160 |
| **vehicle** | 280 | 3,439 | **3,355** | 557 | 1,071 | 86 |
| **walking** | 151 | 1,697 | 128 | **6,959** | 2,343 | 61 |
| **mixed-activity** | 365 | 5,489 | 640 | 3,688 | **10,042** | 187 |
| **bicycling** | 75 | 176 | 78 | 193 | 359 | **944** |

*b) Random Forest + Hidden Markov Model (unit = minutes of behaviour)*

| Ground truth→  Prediction↓ | **sleep** | **sit/stand** | **vehicle** | **walking** | **mixed-activity** | **bicycling** |
| --- | --- | --- | --- | --- | --- | --- |
| **sleep** | **71,253** | 1,973 | 0 | 14 | 18 | 0 |
| **sit/stand** | 1,694 | **43,947** | 551 | 1,464 | 1,594 | 176 |
| **vehicle** | 13 | 697 | **3,967** | 180 | 484 | 12 |
| **walking** | 58 | 1,021 | 177 | **6,856** | 1,452 | 65 |
| **mixed-activity** | 135 | 4,151 | 351 | 3,883 | **11,699** | 158 |
| **bicycling** | 13 | 17 | 10 | 180 | 210 | **1,041** |

1. Difference *(unit = minutes of behaviour)*

| Ground truth→  Prediction↓ | **sleep** | **sit/stand** | **vehicle** | **walking** | **mixed-activity** | **bicycling** |
| --- | --- | --- | --- | --- | --- | --- |
| **sleep** | **5,347** | -1,114 | -22 | -3 | -10 | -13 |
| **sit/stand** | -4,695 | **6,030** | -282 | 302 | -22 | 16 |
| **vehicle** | -267 | -2,742 | **612** | -378 | -587 | -74 |
| **walking** | -93 | -677 | 50 | **-104** | -891 | 4 |
| **mixed-activity** | -231 | -1,339 | -289 | 195 | **1,657** | -29 |
| **bicycling** | -63 | -159 | -69 | -13 | -149 | **97** |
